# Genomic diversity of multidrug-resistant *Rhodococcus equi*: novel sequence types, pangenome architecture, and phylogenomic evolution

**DOI:** 10.1128/aem.02486-25

**Published:** 2026-06-04

**Authors:** Bibek Lamichhane, Ajran Kabir, Alexis A. Adams, Logan Burns, Beth Johnson, Brett Sponseller, Yosra A. Helmy

**Affiliations:** 1 Department of Veterinary Science, Martin-Gatton College of Agriculture, Food and Environment, University of Kentucky 4530, Lexington, Kentucky, USA; 2 College of Veterinary Medicine, Lincoln Memorial University 3747, Harrogate, Tennessee, USA; 3 Division of Lab Services, Kentucky Department for Public Health 98263, Frankfort, Kentucky, USA; Universidad de los Andes, Bogotá, Colombia

**Keywords:** *Rhodococcus equi*, AMR, virulence, horses, WGS, MDR, pangenome, phylogenomic

## Abstract

*Rhodococcus equi* is a major cause of life-threatening pneumonia in foals under 6 months of age and an opportunistic pathogen in immunocompromised humans. Although common in soil and equine farm environments, its evolutionary dynamics, virulence architecture, and pathways of antimicrobial resistance (AMR) emergence remain poorly understood. This study integrated phenotypic characterization and whole-genome sequencing of 46 *R. equi* isolates from horses submitted for necropsy (*n* = 45) and equine fecal samples (*n* = 1) in Kentucky between January 2022 and December 2023 to define their resistance landscape, virulence potential, and population structure. A high burden of multidrug-resistant (MDR) strains was identified (32.6%), including resistance to rifamycin, macrolides, tetracyclines, sulfonamides, and cephalosporins. Nearly all isolates (97.8%) formed robust biofilms, survived intracellularly in macrophages, and carried key virulence determinants (*vapA*, *vapH*, *iupT*). Genomic analysis identified 22 multilocus sequence types (MLSTs), including 20 previously unreported novel sequence types (STs), indicating substantial lineage expansion. Pangenome analysis revealed a large accessory genome and limited core gene conservation; phylogenomic analysis revealed clustering with isolates from humans and other animal hosts, suggesting shared evolutionary ancestry and potential cross-species transmission. These findings highlight *R. equi* as a One Health concern at the animal-environment-human interface. The discovery of numerous novel STs, together with widespread MDR phenotypes and conserved virulence determinants, underscores the need for strengthened antimicrobial stewardship, enhanced genomic surveillance, and development of alternative therapeutic strategies. This study provides critical insights into the epidemiology, AMR emergence, and evolutionary dynamics of *R. equi* circulating within equine farm systems and its implications for both equine and human health.

**IMPORTANCE:**

*Rhodococcus equi* is a bacterium commonly found in soil and around horse farms. While it is part of the natural environment, it can cause serious diseases, including life-threatening pneumonia in young foals and severe infections in people with weakened immune systems. Despite its importance, much remains unknown about how this pathogen spreads, evolves, and develops resistance to antibiotics. In this study, we combined laboratory testing with genome sequencing of *R. equi* obtained from necropsied horses to better understand how these bacteria resist antibiotics and form protective biofilms that make infections difficult to treat. Our genomic analyses revealed many previously unrecognized genetic lineages and showed that horse-associated strains share close evolutionary links with isolates from humans and other animals. Together, these findings highlight *R. equi* as an emerging concern and a potential public health risk, underscoring the importance of responsible antimicrobial use and expanded genomic surveillance to safeguard equine and human health.

## INTRODUCTION

*Rhodococcus equi* is a non-motile, Gram-positive, soil-borne bacterium that primarily causes pneumonia in foals under 6 months of age (1). It was first discovered in the early 20th century and has since remained a major contributor to substantial economic losses in the equine breeding industry worldwide (2). *R. equi* is abundant in farm environments, particularly in pasture soils, where its persistence facilitates both environmental exposure and the acquisition and dissemination of antimicrobial resistance (AMR) determinants (3). Young foals are exposed to virulent *R. equi* from the earliest day of life and become infected by inhaling contaminated dust particles; however, contact with nasal discharge or feces from infected animals can also transmit infection (1).

Once inhaled, *R. equi* infects and replicates within cells of the monocyte–macrophage lineage, especially alveolar macrophages, where it induces necrotic cell death and triggers a strong inflammatory response. This process leads to pulmonary abscessation and the development of subacute to chronic pyogranulomatous bronchopneumonia in foals (4). Clinical signs include fever, coughing, lethargy, tachycardia, and increased respiratory effort, with occasional diarrhea. In addition to respiratory disease, *R. equi* can cause extrapulmonary disorders such as lymphadenitis, polysynovitis, enterotyphlocolitis, and peritonitis (5). Although foals represent the most susceptible population, immunocompromised adult horses may develop disease as well (6).

Beyond equine hosts, *R. equi* infects multiple animal species, including pigs, cattle, and companion animals, where granulomatous pneumonia and lymphadenitis are common manifestations (7). Importantly, *R. equi* is also an opportunistic zoonotic pathogen capable of causing severe disease in immunocompromised humans. Infection usually occurs through direct or indirect contact with infected animals, highlighting the pathogen’s One Health relevance (8). In humans, the disease most often presents as cavitary pneumonia although extrapulmonary infections, including lymphadenitis, brain abscesses, and soft tissue infections, have also been reported (9).

Accurate determination of disease incidence is difficult because diagnosis is often presumptive and based largely on clinical signs. Morbidity and mortality rates vary depending on the infection severity and timing of diagnosis, with pulmonary ultrasonography for abscess detection serving as the mainstay diagnostic tool (4). Currently, no effective vaccines are available for the prevention of infections, and the treatment primarily relies on dual antimicrobial therapy using rifampin in combination with one of the macrolides (such as azithromycin and erythromycin) (10). However, the indiscriminate use of antibiotics to treat subclinical infections without proper diagnostic confirmation has contributed to increased AMR, potentially reducing the clinical efficacy of antibiotics and increasing foal mortality (11). The rising incidence of antibiotic resistance in *R. equi* presents significant treatment challenges, highlighting the necessity for extensive surveillance and the development of alternative therapeutic approaches (2, 12, 13).

This study provides an extensive molecular characterization of *R. equi* isolates obtained from necropsied horses in central Kentucky. By integrating phenotypic virulence traits and antimicrobial susceptibility patterns with whole-genome sequencing (WGS)-based analysis of AMR and virulence determinants, we provide high-resolution insights into population structure, evolutionary relationships, and transmission dynamics. Our findings identify diverse and previously unreported bacterial lineages circulating within the equine population and reveal genomic similarities between equine and human isolates. These results highlight the need for integrated surveillance efforts across human, animal, and environmental domains for risk assessment, infection control, and evidence-based treatment strategies.

## MATERIALS AND METHODS

### Isolation and identification of *R. equi* from the clinical samples

Samples were initially collected from 2,182 horses exhibiting symptoms related to the respiratory and gastrointestinal tracts and submitted for necropsy to the University of Kentucky Veterinary Diagnostic Laboratory (UKVDL) between January 2022 and December 2023. Necropsy specimens were obtained from multiple organs, including the lungs, small intestine, liver, colon, and any other organs displaying gross lesions. The surfaces of the necropsy specimens were sterilized by searing with sterile blades, and the samples were swabbed onto Tryptic soy agar plates (TSA) containing 5% sheep blood (Hardy Diagnostics, CA, USA), Eosin methylene blue (EMB) agar, and Columbia agar (Hardy Diagnostics, CA, USA) containing colistin/nalidixic acid with 5% sheep blood. The inoculated plates were incubated at 37°C for 24 h under microaerobic conditions, followed by an additional 24 h of aerobic incubation at the same temperature. A total of 46 *R. equi* isolates were initially identified based on their characteristic small, smooth, and shiny colony morphology and subsequently confirmed by matrix-assisted laser desorption-ionization time-of-flight mass spectrometry (MALDI-TOF MS) using the Biotyper software (version 4.0; Bruker Scientific, CA, USA). The identity of these isolates was further validated by PCR for the presence of the virulence-associated *vapA* gene (File S1).

### Antibiotic susceptibility test

The antimicrobial susceptibility testing (AST) was performed using the broth microdilution method with a Sensititre Equine EQUIN2F Vet AST plate (ThermoFisher, NY, USA) following the guidelines of the Clinical and Laboratory Standards Institute (CLSI M100: Performance Standards for Antimicrobial Susceptibility Testing 2024) and the manufacturer’s instructions (14, 15). Each EQUIN2F Plate consists of 18 different antimicrobials, including amikacin (AMI; 2–32 µg/mL), ampicillin (AMP; 0.25–16 µg/mL), cefazolin (FAZ; 1–8 µg/mL), chloramphenicol (CHL; 4–32 µg/mL), clarithromycin (CLA; 0.25–8 µg/mL), doxycycline (DOX; 0.12–8 µg/mL), erythromycin (ERY; 0.25–8 µg/mL), gentamicin (GEN; 1–8 µg/mL), imipenem (IMI; 1–8 µg/mL), minocycline (MIN; 0.12–2 µg/mL), oxacillin (OXA+; 0.25–4 µg/mL), penicillin (PEN; 0.12–8 µg/mL), rifampin (RIF; 1–4 µg/mL), tetracycline (TET; 0.25–8 µg/mL), and trimethoprim/sulfamethoxazole (SXT; 0.5/9.5–4/76 µg/mL).

The bacterial isolates were prepared for AST following the manufacturer’s instructions. Briefly, 10 µL of a 0.5 McFarland suspension (OD_600_ = 0.08–0.1) of overnight-grown *R. equi* isolates prepared in distilled water were mixed with Sensititre Mueller-Hinton broth (MH; ThermoFisher, NY, USA), and 50 µL of the suspension was transferred to each well of the 96-well plates and incubated at 37°C. The minimum inhibitory concentrations (MIC) readings were taken after 18 h of incubation using the Sensititre plate reading system. Due to the absence of specific interpretation criteria for antibiotic resistance in *R. equi*, the MICs of the isolates were interpreted using MIC interpretation criteria for human-associated *Staphylococcus aureus* as previously described (6, 16). The antibiotics used for this study, along with the MIC breakpoints according to CLSI, are described in Table S1.

### Biofilm formation assay

The quantification of the biofilm-forming ability of *R. equi* isolates was done as described previously, with minor modifications (15, 17–19). Briefly, *R. equi* isolates were grown aerobically in MH broth (Difco, NY, USA) at 37°C for 24 h with continuous shaking at 200 rpm. After 24 h, 100 µL of each diluted *R. equi* isolate (OD_600_: 0.05; 1 × 10^7^ CFU/mL) was transferred to each well of a flat-bottom 96-well microtiter plate and incubated at 37°C for 48 h without shaking. The planktonic bacteria were carefully removed, and the adhered biofilm was washed (3×) with 125 µL of distilled water and air dried for 20 minutes. Subsequently, the biofilms were stained with 125 µL of 0.1% crystal violet (CV) solution and incubated at room temperature for 20 min. The plates were then washed (3×) with 125 µL of sterile water and allowed to air-dry for 20 min. The biofilm-bound CV was then solubilized in 125 µL of 30% acetic acid, and the biofilm biomass was measured at the wavelength of 550 nm in a microplate reader (Tecan, NC, USA). *R. equi* strain 103S was used as a positive control, and sterile MH broth was used as a negative control/blank. Two independent experiments were carried out with three replicates for each isolate. The results of the biofilm formation of each isolate were categorized as weak, moderate, and strong biofilm producers compared to the optical density of the negative control/blank (OD_c_) as previously described (17). The biofilm-forming ability of the *R. equi* isolates was determined by comparing the optical density of the sample (OD_sample_, OD_s_) with the OD of the control (OD_control_, OD_c_). The OD_c_ was calculated by adding the mean absorbance of the negative control (NC) with three times the standard deviation (SD); (OD_c_ = mean absorbance of NC + 3 SD). The results of the biofilm were characterized as non-biofilm producers (NPB; OD_s_ ≤ OD_c_), weak biofilm producers (WBP; OD_c_ < OD_s_ ≤ 2 O_Dc_), moderate biofilm producers (MBP; 2OD_c_ < OD_s_ ≤ 4 OD_c_), and strong biofilm producers (SBP; 4OD_c_ < OD_s_).

### Intracellular survival assay in murine macrophages

The intracellular survival of *R. equi* isolates in phagocytic cells was assessed using murine macrophage cells (J774A.1; TIB-67). Briefly, J774A.1 cells (1 × 10^5^ cells) were seeded in 96-well plates and maintained in Dulbecco’s modified Eagle’s medium (DMEM; Gibco, NY, USA) supplemented with 10% fetal bovine serum (FBS; Gibco, NY, USA), 2 mM l-glutamine, 5 mM galactose, 1% penicillin/streptomycin (Pen Strep, Gibco, NY, USA), 0.1 mM non-essential amino acids (NEAA; Gibco NY, USA), and 1 mM sodium pyruvate (Cytiva, MA, USA). The monolayer cells were washed (3×) with phosphate-buffered saline (DPBS; Cytiva, MA, USA) and infected with *R. equi* isolates (3.5 × 10⁶ CFU/mL) at an MOI of 10 in antibiotic and FBS-free DMEM for 4 h at 37°C and 5% CO₂. After incubation, the bacteria were removed, and the cells were washed 3× with DPBS and treated with 150 µg/mL gentamicin for 2 h to eliminate extracellular bacteria, followed by incubation in DMEM containing 10 µg/mL gentamicin for 24 h. Cells were then washed, lysed using 0.1% Triton X-100 (Sigma, MO, USA), 10-fold serially diluted, and plated on MH agar to quantify intracellular bacterial burden (20). Two independent experiments were performed.

### Whole genome sequence analysis

For the WGS of the 46 *R. equi* isolates, the genomic DNA of the pure bacterial colonies was extracted using the Qiagen DNeasy Kit (Qiagen, MD, USA) on a QIAcube Connect instrument (Qiagen, MD, USA). The concentration, integrity, and quality of the extracted DNA were determined using the NanoDrop (Thermo Scientific, NY, USA) and 2100 Bioanalyzer system (Agilent, CA, USA). The genomic library preparation was done using the Illumina DNA Prep Kit (Illumina, CA, USA), and genome sequencing was conducted on the MiSeq Illumina platform, generating 2 × 300 bp-long paired-end reads at a depth of approximately 30× as previously described (21–23). Low-quality reads were trimmed out using Trimmomatic v 0.40 (24), and the quality of the raw sequences was assessed using FastQC v0.12.1 (https://github.com/s-andrews/FastQC). Trimmed reads were assembled using SPAdes v4.2.0 (https://github.com/ablab/spades) with default settings. Assembled contigs were annotated using Prokka v1.14.5 (25, 26). Sequence typing was carried out using Multilocus Sequence Typing (MLST; version 2.22.0), applying the *Rhodococcus* schema available through the PubMLST database (https://pubmlst.org/organisms/rhodococcus-spp). Antimicrobial resistance determinants were screened using ABRicate v1.0 against the CARD database with a minimum coverage of 80% and a minimum identity of 90%. For virulence gene determination, assembled contigs were annotated with Bakta v1.11.4 (https://github.com/oschwengers/bakta). Annotated protein FASTA files were screened against the virulence factor database (VFDB) core database of protein sequences (https://www.mgc.ac.cn/cgi-bin/VFs/v5/main.cgi) using DIAMOND v 2.1.16 (https://github.com/bbuchfink/diamond).

Pangenome analysis was performed with 280 *R. equi* genomes retrieved from the NCBI Assembly database (GenBank collections) via the NCBI datasets Genome portal (Genome—NCBI—NLM) (File S1). Prokka-generated GFF files were processed using Roary v3.13.0 (27, 28) to construct and analyze the pangenome. Single-nucleotide polymorphism (SNP)-based distances were generated in Snippy v4.6.0 (https://github.com/tseemann/snippy) to obtain the core SNP alignment. A phylogenetic tree was then inferred using CLC Genomics Workbench 24 using the maximum likelihood method with 1,000 bootstrap replicates. For the accessory genome phylogeny, the output from Roary was used. Both phylogenetic trees were visualized using iTOL (https://itol.embl.de/upload.cgi).

### Molecular detection of virulence and antibiotic-resistance genes

Polymerase chain reaction (PCR) was conducted to detect different virulence and antimicrobial resistance genes (ARGs) of *R. equi* isolates to validate the WGS findings. DNA extraction from each sample was performed using the boiling method as previously described (29, 30). Briefly, a single and isolated colony of *R. equi* grown on TSA + 5% sheep blood (Hardy Diagnostics, CA, USA) at 37°C for 48 h was mixed with 100 μL of nuclease-free water (Cytiva, MA, USA), followed by boiling for 10 min and immediate chilling on ice for 10 min. The mixture was then centrifuged at 5,000 rpm for 10 min, and the supernatants containing the DNA were collected and stored at −20°C for future use. The amplification of the DNA was done with 12.5 µL of GoTaq Green Master Mix (Promega, WI, USA), 2 µL of the template DNA, 10 µM of each of the forward and reverse primers (Thermo Fisher, NY, USA) and adjusted to a final reaction volume of 25 µL in nuclease-free water. For the PCR, initial denaturation was performed at 94°C for 3 min, followed by 35 continuous cycles of denaturation at 95°C for 45 s, annealing at an optimal temperature (*T*_A_) for individual primers, extension at 72°C for 1 min, and final extension at 72°C for 4 min. All the PCR amplifications were done in a Bio-Rad thermal cycler (Bio-Rad, USA), and the final PCR products were visualized in 1% agarose gel containing 0.2% ethidium bromide and photographed using a gel documentation system (Bio-Rad, CA, USA). The oligonucleotide sequences with respective amplicon sizes for each gene used are described in Tables S2 and S3.

### Statistical analysis

All statistical analyses were performed using R Studio (R version 4.5.2) and GraphPad Prism version 10. Associations between categorical variables, including the presence or absence of AMR and virulence genes, were evaluated using Fisher’s exact test. Correlation analysis was conducted using Pearson’s correlation coefficient to assess the strength and direction of relationships between phenotypic antimicrobial susceptibility results, WGS-detected AMR determinants, virulence gene profiles, biofilm formation, and intracellular survival. A *P*-value of <0.05 was considered statistically significant for all analyses.

## RESULTS

### Prevalence of *R. equi* in necropsied horses

Out of the 2,182 samples collected, 46 *R. equi* isolates were recovered from 46 individual horses, demonstrating the overall prevalence of 2.1%. Among the 46 horses, the highest prevalence (80.4%) was observed in foals under 6 months of age (37/46, CI: 68.7%–90.7%; *P*-value: <0.0001) and the lowest prevalence was represented by a single colt of 3-4 years of age (2.2%; 1/46; CI: 0.1%–11.3%; *P*-value: <0.0001). Similarly, a higher prevalence of 52.8% was recorded in females (24/46; CI: 38.1%–65.9%; *P*-value: <0.0001) aged 0 to 6 months (61.8%; *n* = 21) and from lung tissues (52.6%; CI: 38.2%–65.9%; *P*-value: <0.0001) (Table S4).

### Antimicrobial susceptibility of the isolated *R. equi*

The antimicrobial susceptibility results demonstrated that 100% (*n* = 46/46) of the *R. equi* isolates were resistant to cefazolin and penicillin, followed by oxacillin (97.8%; *n* = 45/46) and rifampin (34.8%; *n* = 16/46). Furthermore, 26.1% (*n* = 12/46) of the isolates demonstrated resistance to clarithromycin and erythromycin; 17.4% (*n* = 8/46) to tetracycline; 15.2% (*n* = 7/46) to trimethoprim/sulfamethoxazole; and 6.5% (*n* = 3/46) of the isolates demonstrated resistance to chloramphenicol. All the isolates (100%, *n* = 46/46) were sensitive to amikacin, gentamicin, imipenem, and minocycline (Table 1). Similarly, 32.6% (*n* = 15/46) of the isolates were resistant to at least one antibiotic in three or more antimicrobial classes and were therefore classified as multidrug-resistant (MDR). Two *R. equi* isolates, R4 and R18, demonstrated the highest level of MDR with resistance to 7 classes of antibiotics (cephalosporins, amphenicols, macrolides, penicillin, rifamycin, tetracyclines, and sulfonamides), followed by R14, R21, R26, R31, and R37, which were MDR to 6 classes of antibiotics (cephalosporins, macrolides, penicillin, rifamycin, tetracyclines, and sulfonamides) (Table 2).

**TABLE 1:** Phenotypic antimicrobial sensitivity profile of 46 *R. equi* isolated from necropsied horses

Class of antimicrobials	Antimicrobial^*a*^	Number of positive *R. equi* isolates (*n* = 46)		
		R	I	S
Penicillin	Penicillin	46 (100%)	0	0
	Ampicillin	46 (100%)	0	0
Cephalosporins	Cefazolin	46 (100%)	0	0
Amphenicol	Chloramphenicol	3 (6.5%)	18 (39.1%)	25 (54.3%)
Aminoglycosides	Amikacin	0	0	46 (100%)
	Gentamicin	0	0	46 (100%)
Tetracyclines	Doxycycline	0	1 (2.2%)	45 (97.8%)
	Minocycline	0	0	46 (100%)
	Tetracycline	8 (17.4%)	1 (2.2%)	37 (80.4%)
Carbapenems	Imipenem	0	0	46 (100%)
Sulfonamide	Trimethoprim/ sulfamethoxazole	7 (15.2%)	0	39 (84.8%)
Macrolides	Clarithromycin	12 (26.1%)	0	34 (73.9%)
	Erythromycin	12 (26.1%)	0	34 (73.9%)
Rifamycin	Rifampin	16 (34.8%)	0	30 (65.2%)

^*a*^ Amikacin (AMI), ampicillin (AMP), cefazolin (FAZ), chloramphenicol (CHL), clarithromycin (CLA), doxycycline (DOX), erythromycin (ERY), gentamicin (GEN), imipenem (IMI), minocycline (MIN), penicillin (PEN), rifampin; (RIF), tetracycline (TET), and trimethoprim/sulfamethoxazole (SXT).

**TABLE 2:** Multidrug resistance (MDR) profiles of *R. equi* isolated from horses

Sample ID	Number of resistance classes	MDR classes	MDR phenotypes
R1	4	Cephalosporins, macrolides, penicillins, rifamycin	FAZ, CLA, ERY, OXA+, PEN, RIF
R4	7	Cephalosporins, amphenicols, macrolides, penicillin, rifamycin, tetracyclines, sulfonamides	FAZ, CHL, CLA, ERY, OXA+, PEN, RIF, TET, SXT
R5	6	Cephalosporins, amphenicols, macrolides, penicillin, rifamycin, tetracyclines	FAZ, CLA, ERY, OXA+, PEN, RIF, TET
R6	5	Cephalosporins, amphenicols, macrolides, penicillin, rifamycin	FAZ, CLA, ERY, OXA+, PEN, RIF
R14	6	Cephalosporins, macrolides, penicillin, rifamycin, tetracyclines, sulfonamides	FAZ, CLA, ERY, OXA+, PEN, RIF, TET, SXT
R18	7	Cephalosporins, amphenicols, macrolides, penicillin, rifamycin, tetracyclines, sulfonamides	FAZ, CHL, CLA, ERY, OXA+, PEN, RIF, TET, SXT
R21	6	Cephalosporins, macrolides, penicillin, rifamycin, tetracyclines, sulfonamides	FAZ, CLA, ERY, OXA+, PEN, RIF, TET, SXT
R26	6	Cephalosporins, macrolides, penicillin, rifamycin, tetracyclines, sulfonamides	FAZ, CLA, ERY, OXA+, PEN, RIF, TET, SXT
R31	6	Cephalosporins, macrolides, penicillin, rifamycin, tetracyclines, sulfonamides	FAZ, CLA, ERY, OXA+, PEN, RIF, TET, SXT
R32	4	Cephalosporins, macrolides, penicillin, rifamycin	FAZ, CHL, OXA+, PEN, RIF
R36	4	Cephalosporins, macrolides, penicillin, rifamycin	FAZ, CLA, ERY, OXA+, PEN, RIF
R37	6	Cephalosporins, macrolides, penicillin, rifamycin, tetracyclines, sulfonamides	FAZ, CLA, ERY, OXA+, PEN, RIF, TET, SXT
R41	3	Cephalosporins, penicillin, rifamycin	FAZ, OXA+, PEN, RIF
R42	3	Cephalosporins, penicillin, rifamycin	FAZ, OXA+, PEN, RIF
R48	5	Cephalosporins, amphenicols, macrolides, penicillin, rifamycin	FAZ, CHL, CLA, ERY, PEN, RIF
**Total MDR prevalence**			32.6%

### Biofilm formation

To evaluate the ability of the *R. equi* isolates to form biofilms, we used the CV staining assay. Among the 46 *R. equi* isolates, 97.8% (*n* = 45/46) were found to be biofilm producers. The prevalence of SBP was 28.3% (13/46; CI: 17.32%–42.55%; *P* < 0.0001), MBP was 19.6% (9/46; CI: 10.65%–33.17%; *P* < 0.0001), and WBP was 50% (23/46; CI: 36.19%–65.88%; *P* < 0.0001) (Table 3). The highest biofilm-producing ability was found in isolate R36, followed by isolates R32, R41, R25, and R4. Isolate R14 did not produce any biofilms (Table S5).

**TABLE 3:** The table represents the descriptive analysis for the prevalence of *R. equi* isolates with different biofilm-producing abilities.^*a*^

Isolate characteristic	Number of *R. equi* isolates (*n* = 46)	Prevalence (%)	Upper CI (%)	Lower CI (%)	*P*-value
Strong biofilm producers (SBP)	13	28.3	42.55	17.32	<0.0001***
Moderate biofilm producers (MBP)	9	19.6	33.17	10.65	
Weak biofilm producers (WBP)	23	50.0	63.88	36.12	
No biofilm producers (NBP)	1	2.2	11.34	0.11	
**Total prevalence**		97.8%			

^*a*^ The isolates were divided into four categories based on their ability to form biofilms. SBP, strong biofilm producers; MBP, moderate biofilm producers; WBP, weak biofilm producers; NBP, no biofilm producer.

### Survival of *R. equi* isolates in murine macrophages

The ability of the *R. equi* isolates to survive within the macrophages was evaluated using a gentamicin protection assay in the murine macrophage (J774.A) cell line, with the invasive, highly pathogenic *R. equi* 103S strain being used as a positive control. Among the 46 clinical isolates and 1 invasive control strain *R. equi* 103S, isolate R36, an SBP *R. equi* demonstrated increased replication and persistence inside the macrophages like that of the *R. equi* 103S (9.14-fold higher than the initial infection). This isolate also harbored 100% of the tested virulence genes (*vapA, vapB, vapC, vapD, vapH, virR, virS, iupS*, and *iupT*) required for intra-phagocytic persistence. Similarly, isolates R4 (an MDR and SBP isolate that harbored *vapA, vapB, vapC, vapD, vapH*, and *iupS* virulence genes) and R17 (SBP; harbored 88.9% of the virulence determinants *vapA, vapC, vapD, vapH, virR, virS, iupS*, and *iupT*) also exhibited strong survival and replication within the macrophages with the intracellular bacterial load 4.57-fold higher than the initial infection. Additionally, 6 isolates demonstrated more than 1-fold increase (R3, 2.43-fold; R7, 1.14-fold; R21, 1.57-fold; R22, 1.14-folds; R27, 1.57-fold; and R31, 2.14-fold) in bacterial persistence inside the cells compared to the initial infection, indicating the ability of the isolates to increase their ability to replicate, persist, and cause infection (Table S6). These findings indicate wide variation in the ability of the equine-adapted *R. equi* isolates to replicate and survive inside macrophages, primary sites of replication of the bacteria.

### *R. equi* genome assembly, annotation, and MLST

**Fig 1:**
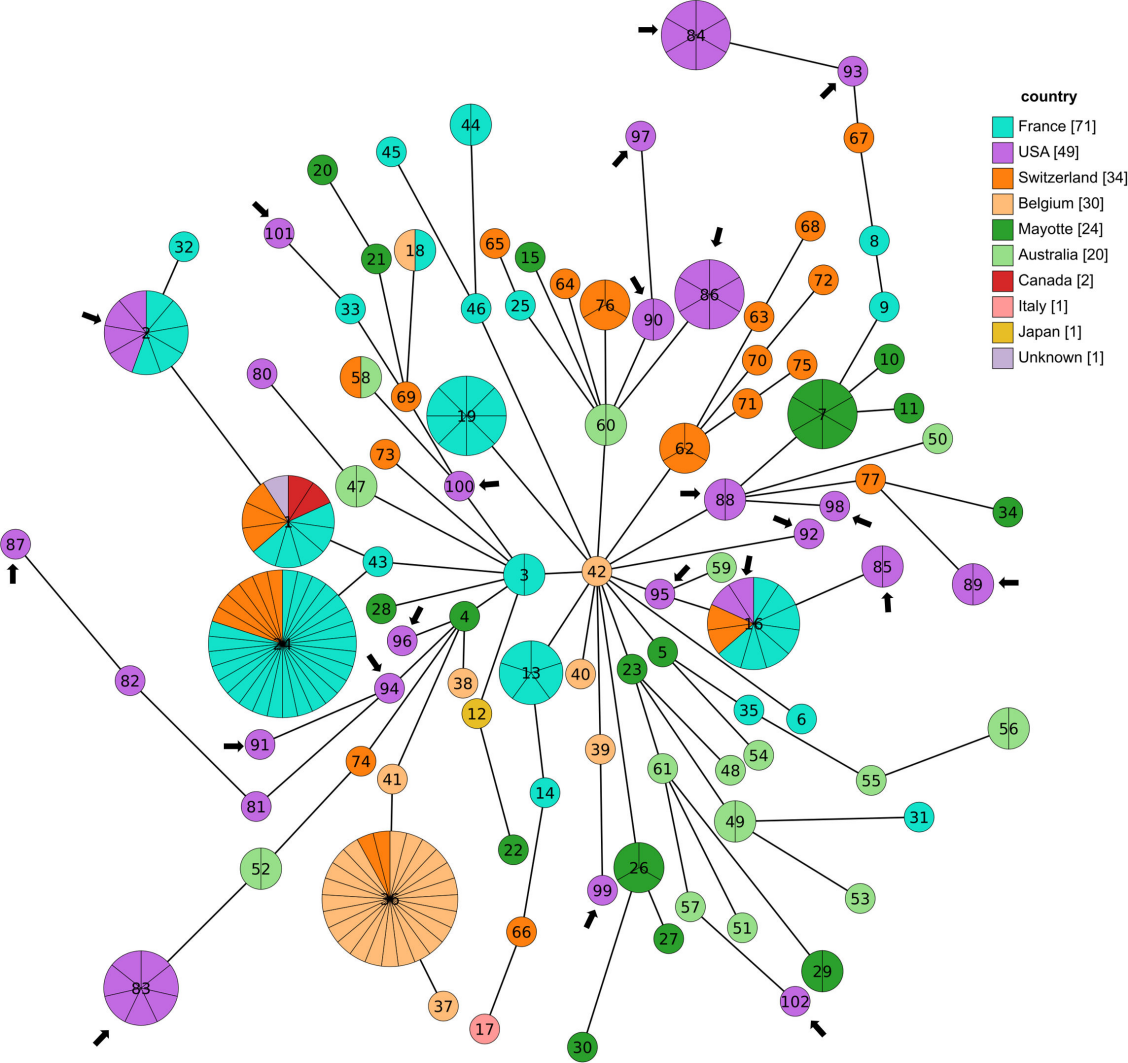
GrapeTree visualization of 46 *R. equi* isolates identified 20 novel STs, along with 2 previously reported STs (ST-2 and ST-16). The GrapeTree illustrates allelic relationships among *R. equi* isolates from the United States (Kentucky, including the 46 isolates from this study) in comparison with global reference STs. Each node represents an individual ST, with node size proportional to the number of isolates within that ST. Each segment in the node pie chart represents an individual isolate. Node colors indicate the geographic origin of the sequence types. Arrows indicate isolates and STs identified in the current study.

The WGS analysis of the 46 *R. equi* isolates collected from horses yielded high-quality genome assemblies for downstream investigation. The mean assembly length was 5,188,088 bp with the genome sizes ranging from 5.0 to 5.6 Mbp. The GC content across all the isolates showed minimal variations, with the mean GC content of 68.7% and spanning a narrow range of 68.6%–68.9% (File S1). However, the continuity of the assembly of the genomes varied between the isolates. Notably, most of the isolates assembled into less than 50 contigs, indicating good-quality draft assemblies. The MLSTs of 46 *R. equi* isolates were determined using a scheme based on the allelic variations in seven housekeeping genes (*adk, gapdh, icl, mdh, recA, rpoB,* and *tpi*). The MLST revealed a substantial allelic diversity among the 46 assemblies of *R. equi* isolates. We identified 6 isolates belonging to two previously reported sequence types (STs): ST-2 (*n* = 4 isolates; R26, R31, R45, and R46) and ST-16 (*n* = 2 isolates; R3 and R25). Interestingly, 40 isolates (R1, R2, R4–R24, R27–R30, R32–R44, R47, and R48) belonged to previously unreported STs. Based on the analysis, we identified two new alleles in *the gapdh, tpi*, and *mdh* genes, three new alleles in the *icl* gene, and one new allele in the *adk* gene. After submission to the PubMLST database, these 20 novel STs were assigned specific ST identifiers starting from ST-83 to ST-102 (Table S7). The detection of a high number of novel STs on MLST indicates that horses in Kentucky harbored a diverse *R. equi* population at the housekeeping gene level. The isolates were found to be highly dispersed across the GrapeTree network when compared with the previously reported STs from the pubMLST database (Fig. 1).

### Pangenome analysis

**Fig 2:**
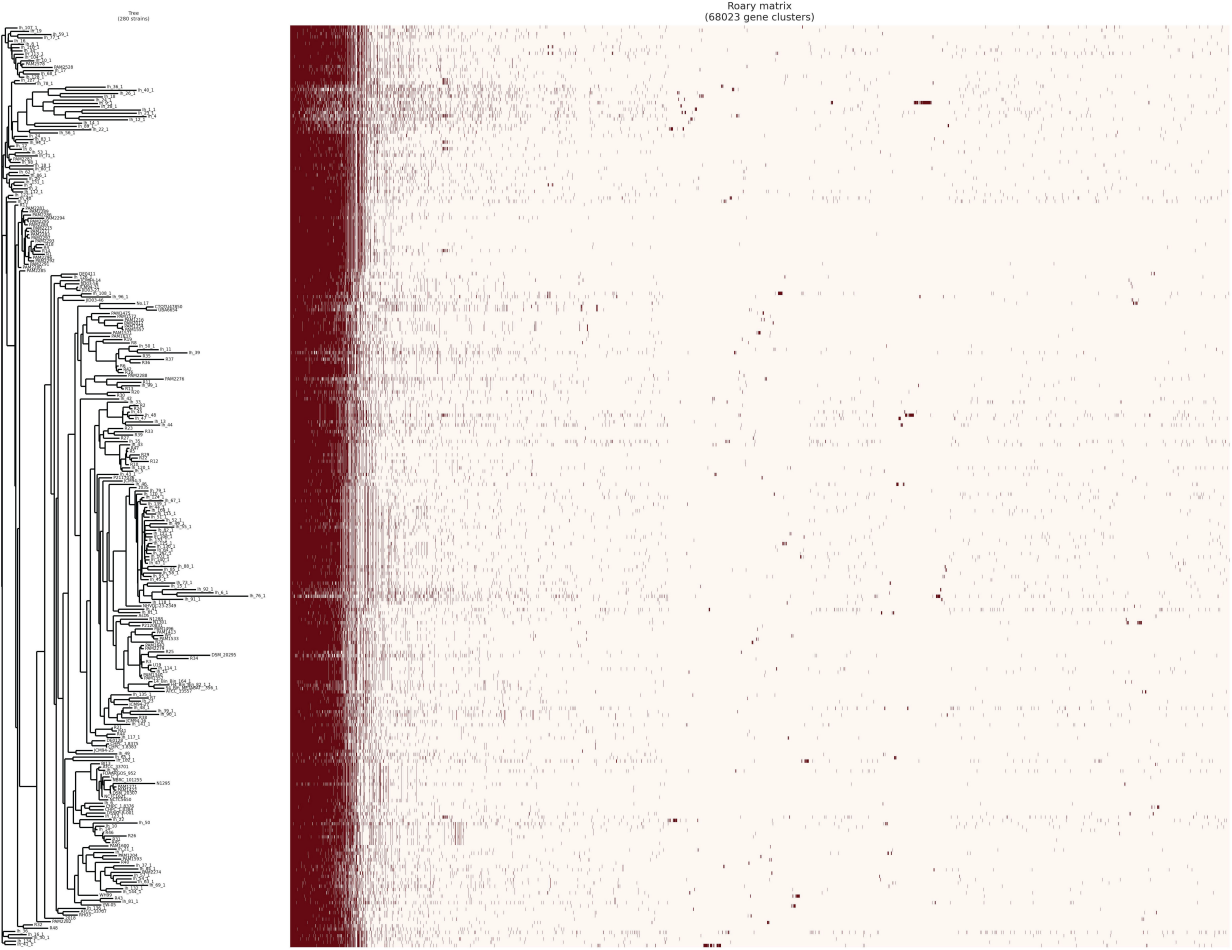
Pangenome gene presence-absence matrix of *R. equi* isolates. Heatmap representation of gene distribution across the analyzed *R. equi* genomes, illustrating the presence and absence patterns of core, accessory, and strain-specific genes. Rows correspond to individual gene clusters, while columns represent individual isolates. Colored cells indicate gene presence, whereas uncolored cells denote gene absence. Hierarchical clustering of both genes and isolates highlights genomic relatedness and potential lineage-specific gene associations, providing insight into patterns of genome conservation, diversification, and putative functional specialization across the population.

The pangenome analysis of the 280 isolates (46 isolates from this study and 234 isolates from NCBI) revealed substantial genomic diversity among the isolates. The average genes per strain in all 46 isolates ranged from 4,700 to 5,000 (File S1). The size of the pangenome showed a near linear increment, and the gene repertoire continuously expanded when more genomes and isolates were added, leading to a total of 68,023 gene clusters. In contrast, the number of conserved genes continuously decreased when new genomes were introduced, stabilizing at around 476 (0.69% of the pangenome) strict core genes and 1,889 (2.78% of the pangenome) soft-core genes (Fig. S1a and b). Interestingly, sharp breaks in the trend at V117 and V201 regions were observed, indicative of the introduction of an unusually divergent genome with reduced gene overlap (Fig. S1c). The accessory genes were dominated by the presence of highly variable genes found in only small subsets of genes, often singletons or strain-specific genes (cloud genes: 62,037; 91% of the pangenome), and a moderate number of the subsets of genes shared by two or more strains (shell genes 3,621; 5.3% of the pangenome). Interestingly, the number of unique genes increased with the expansion of the number of genomes (Fig. S1d), while the number of new genes remained relatively constant, indicating a high level of horizontal gene transfer or environment-specific adaptation. The hierarchical clustering of the 46 isolates with the rest of the 234 genomes tested demonstrates high genomic plasticity, which was dominated by highly heterogeneous accessory genes with sparse and patchy distribution (Fig. 2).

### Phylogenetic analysis and pairwise single-nucleotide polymorphism difference

The core-genome phylogenetic analysis of *R. equi* isolates demonstrated that the majority of the isolates were grouped within the dominant *R. equi* lineage isolated from horses in central Kentucky. However, despite being collected from the same host species, year, and geographical location, isolates R1, R4, R14, R17, R32, and R48 branched independently and did not cluster with the main equine clade, indicating multiple genetically distinct *R. equi* lineages are circulating simultaneously within the equine population of Kentucky.

**Fig 3:**
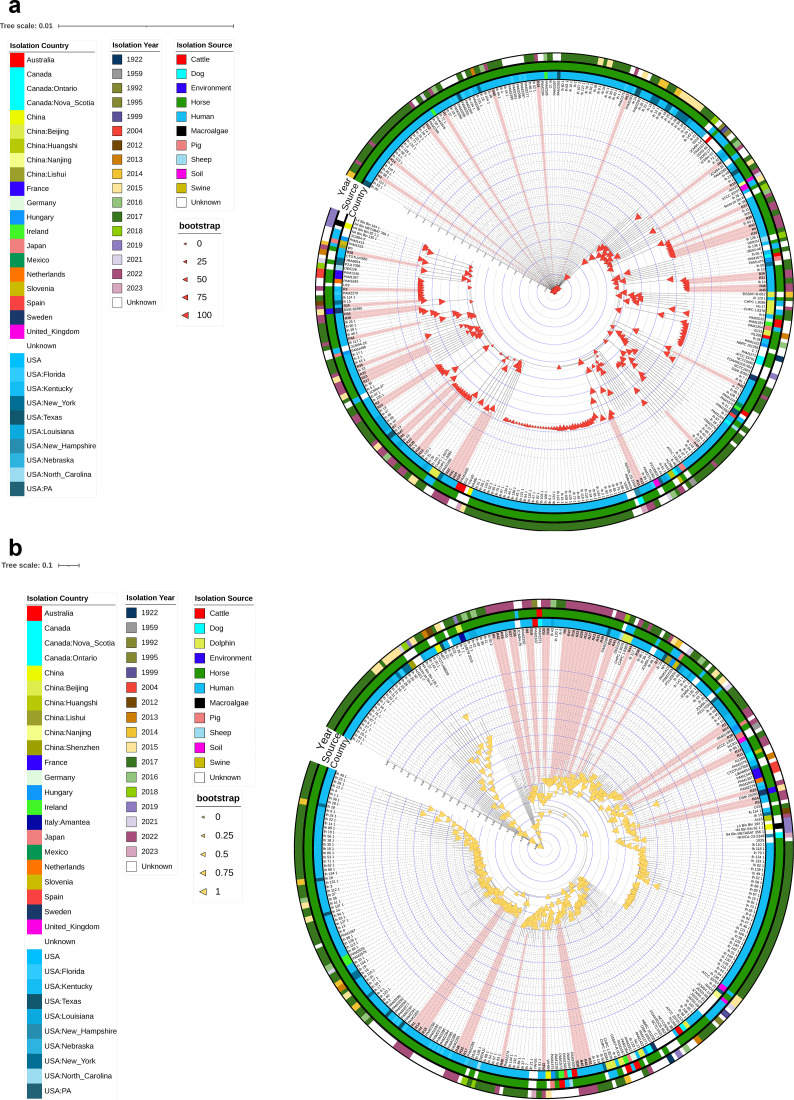
Circular maximum-likelihood phylogeny reconstructed using (**a**) the core genome alignment, demonstrating that most isolates from this study cluster within the dominant equine-associated *R. equi* lineage circulating in central Kentucky. However, several isolates, despite being recovered from the same host species, geographic region, and collection period, form distinct branches and did not cluster within the main equine clade, with a subset of isolates showing close phylogenetic relatedness with non-equine strains, and (**b**) accessory genome alignment of *R. equi* isolates, highlighting greater genomic heterogeneity compared to the core genome with isolates showing more dispersed clustering patterns. Concentric rings surrounding the tree display isolation-associated metadata, year of isolation, sample source, and country of origin.

A small subset of the isolates clustered closely with the isolates originating from humans or pigs, demonstrating that certain lineages of the bacteria can infect both humans and animals and thus have zoonotic significance. For example, the isolate R8 is clustered with the human-derived strain “JCM94-25” within the same clade, while isolates R23, R27, R33, and R39 grouped closely with another human isolate “JCM94-27.” Likewise, isolate R28 clustered within the same clade as two non-equine isolates WY (isolated from a human in China in 2014) and PAM1533 (isolated from pigs in China) (Fig. 3a).

The accessory gene level phylogenetic analysis of 46 *R. equi* isolates revealed a strong clustering of most of the isolates with previously reported *R. equi* from Kentucky, indicating highly similar accessory genes, including mobile genetic elements and environmental adaptation factors. Consistent with the core-genome findings, the isolates R1, R4, R14, R17, R32, and R48 clustered separately from the dominant Kentucky equine clade, suggesting the presence of multiple lineages of circulating accessory genes within Kentucky. Several isolates also clustered within the same clade as non-equine strains originating from diverse geographical regions. For example, isolate R28 not only clustered with WY (human, China) and PAM1533 (pigs, China) at the core genome level but also clustered with PAM1496 isolated from pigs in Hungary at the accessory gene level. Additionally, isolate R43 clustered with WH99 from China and isolates R1, R4, R14, and R18 clustered with PAM2283, PAM2297, and PAM2277 from Florida and PAM2291 from Texas, indicating that some accessory gene lineages circulate in multiple geographical locations and host species (Fig. 3b). This accessory genome structure suggests the circulation of stable, locally adapted lineages with few episodic introductions of accessory gene repertoires within the equine population.

The core-genome single-nucleotide polymorphism (SNP) comparison of all *R. equi* isolates included in the comparative study demonstrated a high level of overall genetic diversity among the circulating isolates. Pairwise SNP differences among the isolates revealed that only 0.6% of the isolates differed by ≤3 SNPs, 0.6% by ≤5 SNPs, and 0.6% by less than 10 SNPs, indicating a small number of highly clonal pairs and more than 99% of the isolates being genetically diverse isolates with no evidence of widespread outbreak of the bacteria (Fig. S2). This cumulative distribution of the genetically diverse isolates further confirmed the absence of the large transmission clusters.

### Genotypic prediction of antimicrobial resistance and virulence

We screened all 46 *R. equi* genome assemblies for AMR determinants using the Comprehensive Antibiotic Resistance Database (CARD). All isolates carried a conserved set of intrinsic rifampin-associated genes (*rpoB2* and *rbpA*), which are well-recognized housekeeping genes that contribute to the basal rifampin resistance. In contrast, acquired ARGs were detected only in a subset of isolates. These included *erm*(46) (macrolide resistance), *aadA9* (aminoglycoside resistance), *tet*(33) (tetracycline resistance), and *sul1* (sulfonamide resistance). Notably, four isolates (R4, R14, R18, and R37) harbored all six ARGs, representing the MDR fraction within the population. The acquired ARGs identified in these isolates, as well as the *erm (46*) genes found in isolates R1, R6, R35, R36, and R48, showed 100% identity and coverage to previously reported resistance genes in CARD. Overall, the AMR profiles derived from the WGS data suggest that Kentucky *R. equi* isolates predominantly possess intrinsic resistance signatures, with acquired resistance detected at low frequency and MDR genotypes present in a small subset of isolates (Table S8).

The WGS predicted the presence of a total of 176 virulence factor genes (VFGs) across 46 *R. equi* isolates from horses. Out of 176 genes, 151 virulence-associated genes (VAGs) were found to be conserved in all the isolates as annotated using the VFDB. Isolates R11, R35, and R36 demonstrated the presence of the lowest number of virulence genes (*n* = 161) (Fig. S3, File S2). Similarly, the population contained the highly conserved VAGs such as *hspR* (heat shock protein transcriptional repressor), *phoP* (possible two-component system response transcriptional positive regulator), *prrA* (two-component system response regulator), *ideR* (iron-dependent repressor and activator), *mbtH* (putative protein for biosynthesis of siderophores), *regX3* (two-component sensory transduction protein), and *adhD* (NDMA-dependent alcohol dehydrogenase for bacterial immune modulation).

### Validation of antibiotic-resistance and virulence determinants

Antimicrobial resistance and virulence genes were validated using PCR. In addition to these targets, several genes not captured in the CARD or VFDB databases were also confirmed through PCR amplification. We identified the oxabeta-lactamase gene *oxa134,* which confers resistance to tetracyclines, glycopeptides, aminoglycosides, and carbapenems, as the most prevalent one and was identified in all the isolates (100%, *n* = 46/46). Similarly, the rifampin/rifamycin resistance gene *rbpA* was detected in 95.6% (*n* = 39/46) of the isolates. This was followed by the aminoglycoside resistance gene *aadA9* and glycopeptide resistance gene *vanW* (84.8%, *n* = 39/46) and sulfonamide resistance *sul1* and macrolide resistance genes *erm*(46) (78.3%, *n* = 36/46). The tetracycline resistance gene *tet (33*) was the least prevalent, being detected in 60.9% of the isolates (*n* = 28/46) (Table 4; Fig. S4a). Plasmid-encoded virulence genes that were not available in the VFDB database were detected through PCR amplification. The virulence plasmid encoding genes responsible for intra-macrophage survival, including *vapA* and *vapH,* were detected in 100% (*n* = 46/46) of the isolates. Other virulence plasmid encoding genes were also highly prevalent, including *vapD* (93.5%, *n* = 43/46), *vapC* (89.1%, *n* = 41/46), followed by *vapB* (84.8%, *n* = 39/46). Similarly, we detected transcriptional regulators of *R. equi* virulence *virS* in 89.1% (*n* = 41/46) and *virR* in 82.6% (*n* = 38/46) of the samples. In addition, iron uptake/siderophore associated genes *iupT and iupS* were detected in 97.8% (*n* = 45/46) and 89.1% (*n* = 41/46) of the isolates, respectively (Table 5; Fig. S4b).

**TABLE 4:** Genotypic detection of antibiotic-resistant determinants in *R. equi* isolates using PCR

Gene	Antimicrobial(s)	Number of positive isolates (*n*)	Prevalence (%)
*oxa134*	Carbapenem	46/46	100
*rbpA*	Rifampin	44/46	95.7
*erm(46)*	Macrolides, lincomycin, streptogramin	39/46	84.8
*aadA9*	Aminoglycosides	39/46	84.8
*vanW*	Glycopeptides (vancomycin)	39/46	84.8
*sul1*	Sulfonamide	36/46	78.3
*tet(33)*	Tetracycline	28/46	60.8

**TABLE 5:** Genotypic detection of virulence associated genes in *R. equi* isolates using PCR

Function	Gene	Number of positive isolates	Prevalence %
Virulence associated protein	*vapA*	46	100.0
	*vapB*	39	84.8
	*vapC*	41	89.1
	*vapD*	43	93.5
	*vapH*	46	100.0
Iron acquisition	*iupS*	41	89.1
	*iupT*	45	97.8
Transcriptional regulators	*virR*	38	82.6
	*virS*	41	89.1

### Correlation of phenotypic traits and genomic AMR and virulence profiles

The comprehensive Pearson’s square correlation and hierarchical clustering analysis integrating phenotypic AST profiles, WGS-detected AMR genes, PCR-confirmed resistance markers, virulence determinants, biofilm phenotypes, and intracellular survival capacity in macrophages revealed multiple strong and biologically coherent positive associations across the data set.

The phenotypic macrolide resistance (erythromycin and clarithromycin) showed a strong positive correlation with resistance to rifampin (*r* = 0.81; *P* < 0.001), tetracyclines, and trimethoprim/sulfamethoxazole (*r* = 0.71; *P* < 0.001). Tetracycline resistance also strongly correlated with trimethoprim/sulfamethoxazole resistance (*r* = 0.71; *P* < 0.001), while a moderate correlation was observed between rifampin and tetracycline resistance (*r* = 0.58; *P* < 0.001). These significant correlations indicate that these phenotypes co-occur frequently within the same isolates, suggesting the presence of an MDR background between the isolates with multiple AMR determinants maintained together. A strong correlation was also detected between phenotypic oxacillin resistance and the PCR detection of the rifampin-associated gene *rbpA* (*r* = 0.70; *P* < 0.001), meaning isolates tend to exhibit broader resistance characteristics in their MDR lineages. In contrast, the presence of *aadA9* (aminoglycoside adenyltransferase) showed a negative correlation with phenotypic resistance to chloramphenicol (*r* = –0.34; *P* < 0.05), macrolides (*r* = –0.30; *P* < 0.05), tetracyclines (*r* = –0.40; *P* < 0.01), and trimethoprim/sulfamethoxazole (*r* = –0.33; *P* < 0.05). Similarly, the correlation analysis between phenotypic AST results and WGS-detected AMR genes further demonstrated strong positive associations between trimethoprim/sulfamethoxazole resistance and *sul1* (*r* = 0.73; *P* < 0.001), tetracycline resistance and *tet*(33) (*r* = 0.63; *P* < 0.001), and macrolide resistance (erythromycin, clarithromycin) with both *sul1* and *tet*(33) (*r* = 0.52; *P* < 0.001). A significant positive correlation was also observed between rifampin resistance and *sul1* and *tet*(33) (*r* = 0.42; *P* < 0.01). A weak positive correlation was noted between rifampin resistance and *erm*(46) (*r* = 0.22; *P* > 0.05). The co-occurrence of multiple resistance genes with macrolide, tetracycline, trimethoprim/sulfamethoxazole, and rifampin resistance further implies that these determinants are frequently carried together within MDR genomic backgrounds or circulating virulence plasmids. The correlation of the phenotypic resistance with the whole-genome prediction of the AMR determinants further validates the WGS results.

**Fig 4:**
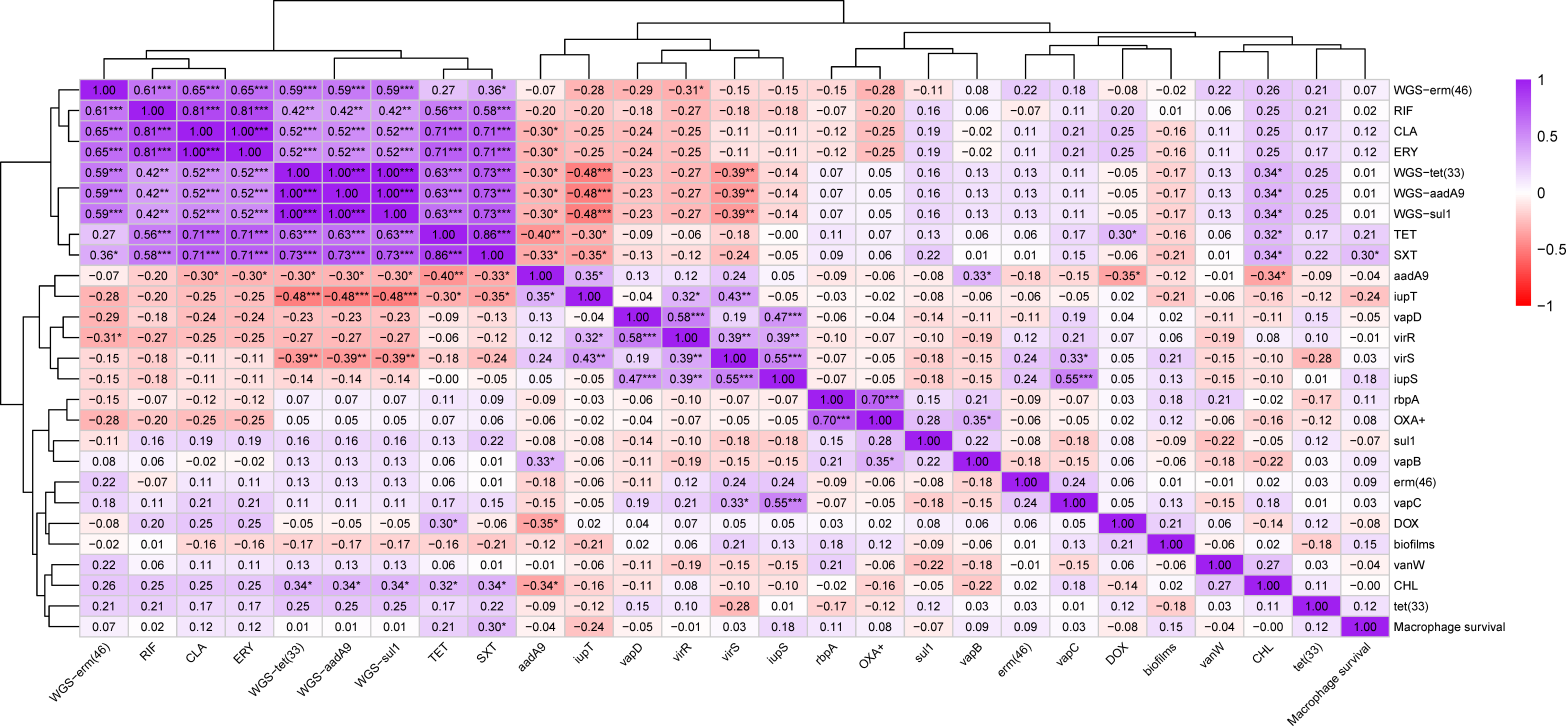
Pearson correlation coefficient heatmap indicating the relationship between the occurrence of phenotypic AMR, biofilm-forming capacity, ability to survive inside macrophages, PCR detection of ARGs, virulence determinants, and whole genome prediction of antibiotic resistance determinants. Significant positive correlations were observed among key AMR phenotypes, which also correlated strongly with corresponding resistance genes identified by PCR and WGS. Virulence-associated genes showed moderate to strong positive associations. Similarly, weak positive correlations were observed between virulence determinants and phenotypic traits such as macrophage survival and biofilm formation. The asterisks with the r-scores (correlation coefficient) represent statistical significance: ***P < 0.001, **P < 0.01, *P < 0.05.

Similarly, we found a strong positive correlation between the molecular detection of virulence determinants *vapD* with *virR* (*r* = 0.58; *P* < 0.001) and *virS* (*r* = 0.19; *P* > 0.05); *virR*, *virS* (*r* = 0.39; *P* < 0.01), *iupS* (*r* = 0.39; *P* < 0.01), and *iupT* (*r* = 0.32; *P* < 0.05). The identification of the virulence plasmid-associated gene *vapA* in 100% of the isolates and the strong and significant correlation between the virulence determinants suggest co-occurrence of the virulence genes, possibly reflecting the coordinated presence of a virulence plasmid harboring these genes. We also observed a weak positive correlation between macrophage survival and virulence determinants *iupS* (*r* = 0.32; *P* > 0.05), which is important for sequestering iron required for survival of the bacteria inside the macrophages. Similarly, the biofilm-forming ability of the isolates demonstrated a weak positive correlation with the prevalence of *vapD* (*r* = 0.02; *P* > 0.05), *virR* (*r* = 0.06; *P* > 0.05), *virS* (*r* = 0.21; *P* > 0.05), and *iupS* (*r* = 0.13; *P* > 0.05). The presence of this positive correlation, although weak, suggests that these virulent determinants may contribute to the ability of the bacteria to form biofilms (Fig. 4).

## DISCUSSION

*R. equi* is considered one of the most common causes of severe pneumonia in foals, often requiring prolonged antimicrobial therapy, leading to substantial economic loss in the equine breeding industry (31). Despite being treated with antibiotics such as macrolides and rifampin, therapeutic failures are common, largely due to emerging AMR and key virulence traits, including the bacterium’s ability to persist within macrophages and form biofilms (2). In addition, the widespread environmental presence, particularly in contaminated soil, creates continuous exposure risks to susceptible foals and contributes to the recurrence and persistence of infection (3). Beyond horses, *R. equi* also infects a wide range of hosts, including cattle, pigs, wild boars, companion animals, and immunocompromised humans (32). The interplay between animal disease, environmental contamination, and human health underscores the critical need for environmental surveillance and antimicrobial stewardship to limit bacterial dissemination and zoonotic transmission (19, 32). To elucidate the status of *R. equi*, our study characterized 46 isolates using phenotypic AMR testing, virulence profiling, and WGS, which provides a comprehensive assessment of their pathogenic potential and transmission dynamics.

The overall prevalence of *R. equi* in this study was 2.1%, which is slightly lower than a previous study, which reported a 3.1% prevalence in nasopharyngeal swabs and 2.6% in nasal swabs collected from healthy horses in India between 2013 and 2015 (33, 34). Differences in the targeted site for *R. equi* detection may be one reason for such variability.

The highest prevalence was seen in foals under 3 months of age (45.7%), followed by 3–6 months of age (26.9%), which is consistent with the susceptibility of young foals to *R. equi* in these age groups (35). The isolation of *R. equi* was highest from the lung tissues (52.5%), reflecting the intracellular nature of the bacteria and its high affinity to alveolar macrophages, consistent with the lungs being recognized as a major predilection site (4). Although the recovery of the bacteria from feces was relatively low (2.2%) (Table S4), horses are known to shed *R. equi* persistently through feces, contributing to environmental contamination and transmission to foals (35).

In this study, 34.8% of the 46 *R. equi* isolates were resistant to rifampin, while 26.1% showed resistance to the macrolides, including clarithromycin and erythromycin (Table 1), with 32.6% displaying the MDR phenotype (Table 2). These rates are slightly higher than those reported in Kentucky (2011–2019) from 256 clinical isolates, where 22.7% of isolates were resistant to rifampin and 15.2% and 14.8% were resistant to erythromycin and clarithromycin, respectively (6). Similarly, another study reported an increase in rifampin resistance from 2.3% to 16.1% and 0.7% to 13.6% for erythromycin in central Kentucky (16). Although variable, both studies reported that resistance rates are lower than those observed in this study, highlighting a concerning trend of rising AMR in *R. equi,* likely due to escalating selection pressures exacerbated by the widespread use of rifampin–macrolide therapy, often administered without bacteriological confirmation or knowledge of resistance phenotypes (36).

Biofilms are one of the major underlying causes of antimicrobial treatment failure and increased disease severity (37). Biofilms enhance bacterial survival by protecting them from environmental stress factors such as UV exposure, desiccation, and nutrient limitation, thereby supporting long-term survival in soil and farm dust (37). In our study, 97.8% of the isolates formed biofilm (Table 3; Table S5). Notably, 100% of the MDR isolates demonstrated moderate to high biofilm formation and strong persistence inside the macrophages. This is consistent with the fact that strong biofilm-producing bacteria demonstrate increased attachment to phagocytic cells (38). This facilitates their persistence within macrophages and promotes the expression of the key virulence determinants such as *vapA*, *vapH*, and the iron-acquisition genes *iupS* and *iupT*, which are essential for intramacrophage replication (39). Once internalized, the expression of virulence plasmid-encoded proteins (*vapA, vapB, vapC, vapD, vapH*) enables *R. equi* to withstand the acidic and hostile environment within macrophages (40). The near-universal biofilm-forming ability, together with the high prevalence of key virulence genes, likely contributes to enhanced persistence and intracellular survival of *R. equi*, promoting macrophage destruction and granuloma formation and ultimately driving the severe respiratory disease seen in foals (39). These findings support the concept that virulence and AMR determinants collectively potentiate the bacterium’s ability to form biofilms and replicate within phagocytic cells, thereby sustaining infection and complicating treatment (41).

The WGS analysis offered insights into evolutionary relationships, transmission dynamics, novel lineages, mobile genetic elements, and broader pangenome structure in our isolates, features that cannot be captured through phenotypic testing alone (42). The WGS analysis revealed a mean assembly length of 5,188,088 bp for the 46 *R. equi* isolates (File S1), which falls within the established genomic range of *R. equi* (5.0–5.5 Mbp; 68.5%–68.9%) (43, 44). The MLST analysis revealed a remarkable allelic diversity with 20 novel STs among 40 out of 46 isolates co-circulating within the equine population in Kentucky, suggesting repeated environmental acquisition rather than clonal expansion. This pattern aligns with sporadic, environmentally driven infection dynamics of *R. equi* rather than outbreak-related transmission (45). Similarly, MLST-based GrapeTree revealed singletons and small USA-specific clusters, indicating locally persisting lineages (Fig. 1) (46). In contrast, many isolates clustered with French, Belgian, Swiss, and Australian STs, indicating long-distance dissemination of strains, likely facilitated by equine movement during international equine-related events (47).

The pangenome analysis revealed high genomic plasticity with only 2.8% strict-core genes and more than 91% variable cloud genes, indicating an open pangenome structure. This is consistent with the fact that the analysis was performed with an extended data set of 280 genomes from diverse geographical locations, hosts, and ecological niches, which resulted in a progressive reduction in the proportion of the core genome. These features are characteristic of *R. equi*, which is frequently exposed to diverse ecological niches and supports ongoing acquisition of genes related to survival under diverse environmental stresses, including novel AMR determinants (48, 49). Interestingly, the core-genome phylogenetic analysis showed that most of the *R. equi* isolates clustered within established equine-associated Kentucky lineages, while several (R1, R4, R14, R32, R48) branched independently (Fig. 3a). This diversity indicates the co-circulation of multiple genetically distinct lineages within the same host population within the same region. The lack of a single dominant clade further suggests that infections likely arose from repeated environmental exposure rather than a single outbreak event (48). Accessory genome comparisons also showed similarities between Kentucky isolates and strains from China, Hungary, and U.S. states such as Florida and Texas, suggesting regional and global movement of certain genotypes, likely driven by horizontal gene transfer and mobile genetic elements (50, 51). The absence of a single dominant clade supports the likelihood that these infections resulted from diverse environmental exposure (48). Moreover, core genome similarities between equine isolates and strains from humans and pigs highlight the cross-host distribution of *R. equi*. Additionally, pairwise SNP distance analysis showing that more than 99% of isolates differed by over 10 SNPs indicates the presence of unrelated strains, supporting environmental acquisition rather than recent horse-to-horse transmission or a single outbreak event (52, 53).

The WGS analysis identified several acquired AMR determinants (*erm (46*), *aadA9*, *tet (33*), and *sul1*) in a subset of isolates. Their restricted yet repeated occurrence within the accessory genome suggests horizontal gene transfer as a likely mechanism of acquisition, and the presence of these genes may confer a survival advantage under repeated antibiotic stress (3). When combined with the high diversity on MLST and core-genome phylogeny, these findings indicate that genetically distinct lineages can gain resistance through shared mobile resistance genes or modules (54). Although the WGS-based AMR predictions were largely supported by PCR (Tables 4 and 5), some genes amplified by PCR were not detected by WGS, most likely due to nonspecific amplification or fragmented gene sequences below WGS annotation thresholds (55). The absence of a well-known, standardized database specifically dedicated to *R. equi* AMR and virulence determinants constituted a major methodological limitation of this study. Similarly, another limitation of this study was the WGS analysis based on draft genome assemblies. This limited our ability to resolve complete plasmid sequences or fully characterize mobile genetic elements carrying ARGs and VFGs. Further studies employing complete genome assembly along with plasmid reconstruction will be essential to elucidate the genomic context, mobility, and transmission dynamics of these elements.

Overall, our findings highlight the emergence of MDR, genetically diverse, and highly virulent *R. equi* lineages circulating in Kentucky. The convergence of strong biofilm formation, conserved virulence determinants, extensive genomic diversity, and phylogenomic links to human isolates underscores the urgent need for integrated genomic surveillance across equine farms, the environment, and human health sectors. Future work should prioritize developing a comprehensive *R. equi* genomic reference database, understanding host immune responses to diverse lineages, evaluating therapeutic alternatives, and monitoring environmental reservoirs to inform evidence-based interventions and thereby reduce disease burden.

### Conclusion

Our findings reveal a rising concern posed by virulent, MDR *R. equi* circulating in equine farm environments in Kentucky with possible One Health implications. The high prevalence of AMR, particularly resistance to clinically important drugs such as rifampin and macrolides, combined with the widespread distribution of virulence determinants, robust biofilm formation, and the ability of isolates to invade and survive within phagocytic cells, underscores the substantial clinical challenges associated with *R. equi* infections in foals. The detection of MDR and virulent lineages with evolutionary linkage to isolates derived from different host species emphasizes the urgent need for comprehensive surveillance programs to monitor the emergence and spread of high-risk *R. equi* strains in equine populations. Given the significant economic impact of *R. equi* on the equine breeding industry, the rise of MDR *R. equi* represents a critical concern for both veterinary and broader health sectors.

Integrating WGS to characterize AMR determinants, virulence profiles, and transmission dynamics across hosts will be essential for guiding targeted infection control strategies and strengthening antimicrobial stewardship. Such genomic and phenotypic surveillance approaches are vital for mitigating the growing burden of *R. equi* on animal health and for informing targeted interventions to protect equine populations and improve disease management in the equine breeding industry, protect vulnerable human populations, and inform One Health-aligned interventions that address a possible risk at the animal-environment-human interface.

## Data Availability

All isolates were submitted to the NCBI Sequence Read Archive under BioProject no. PRJNA1291752. All supporting data have been provided within the article and its supplemental material.

## SUPPLEMENTAL MATERIAL

File S1 aem.02486-25-s0001.xlsx Metadata and genomic information for *R. equi* genomes included in this study.

File S2 aem.02486-25-s0002.xlsx Whole genome analysis of virulence factor genes.

Supplemental figures aem.02486-25-s0003.pdf Fig. S1 to S4.

Supplemental legends aem.02486-25-s0004.docx Descriptive legends for supplemental material.

Supplemental tables aem.02486-25-s0005.docx Tables S1 to S8.

ASM does not own the copyrights to Supplemental Material that may be linked to, or accessed through, an article. The authors have granted ASM a non-exclusive, world-wide license to publish the Supplemental Material files. Please contact the corresponding author directly for reuse.
