# Supplementary material for: Genomic diversity of multidrug-resistant Rhodococcus equi: novel sequence types, pangenome architecture, and phylogenomic evolution
Source: Appl Environ Microbiol. 2026 Jun 4;92(7):e02486-25. doi: 10.1128/aem.02486-25 (PMC13390474; doi:10.1128/aem.02486-25)
Supplement: Supplemental figures — Fig. S1 to S4. [file aem.02486-25-s0003.pdf]

Figure S1a

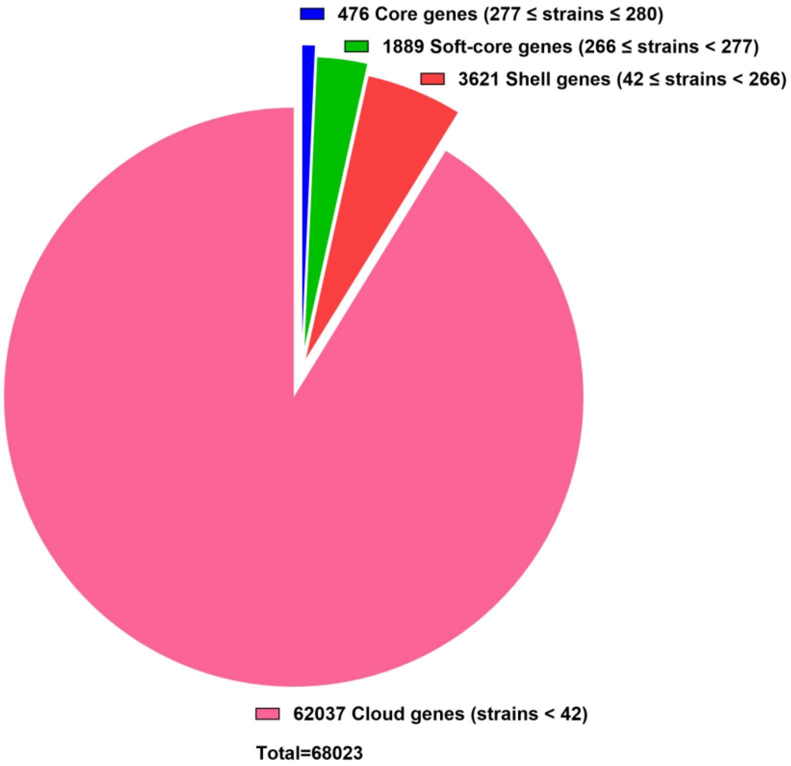

Figure S1b

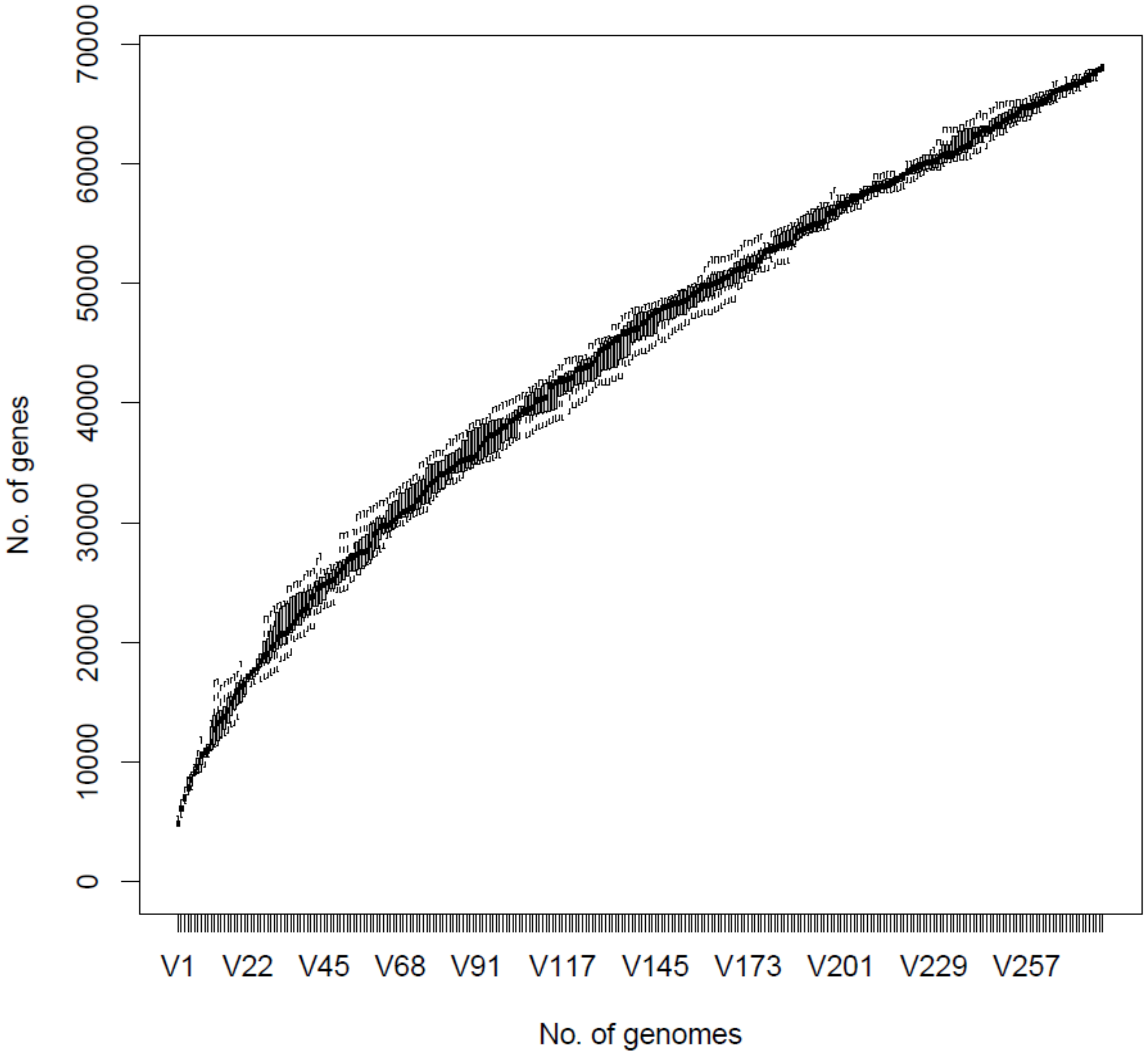

Figure S1c

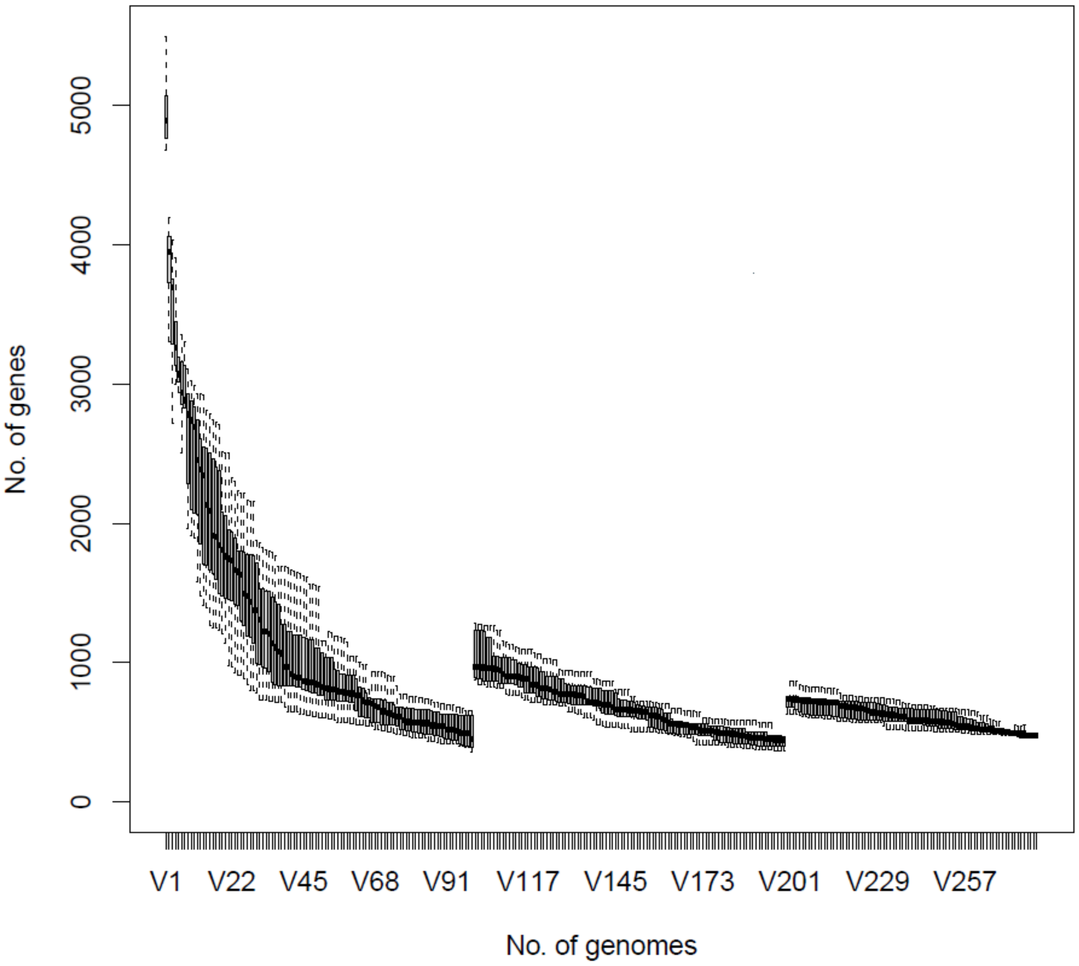

Figure S1d

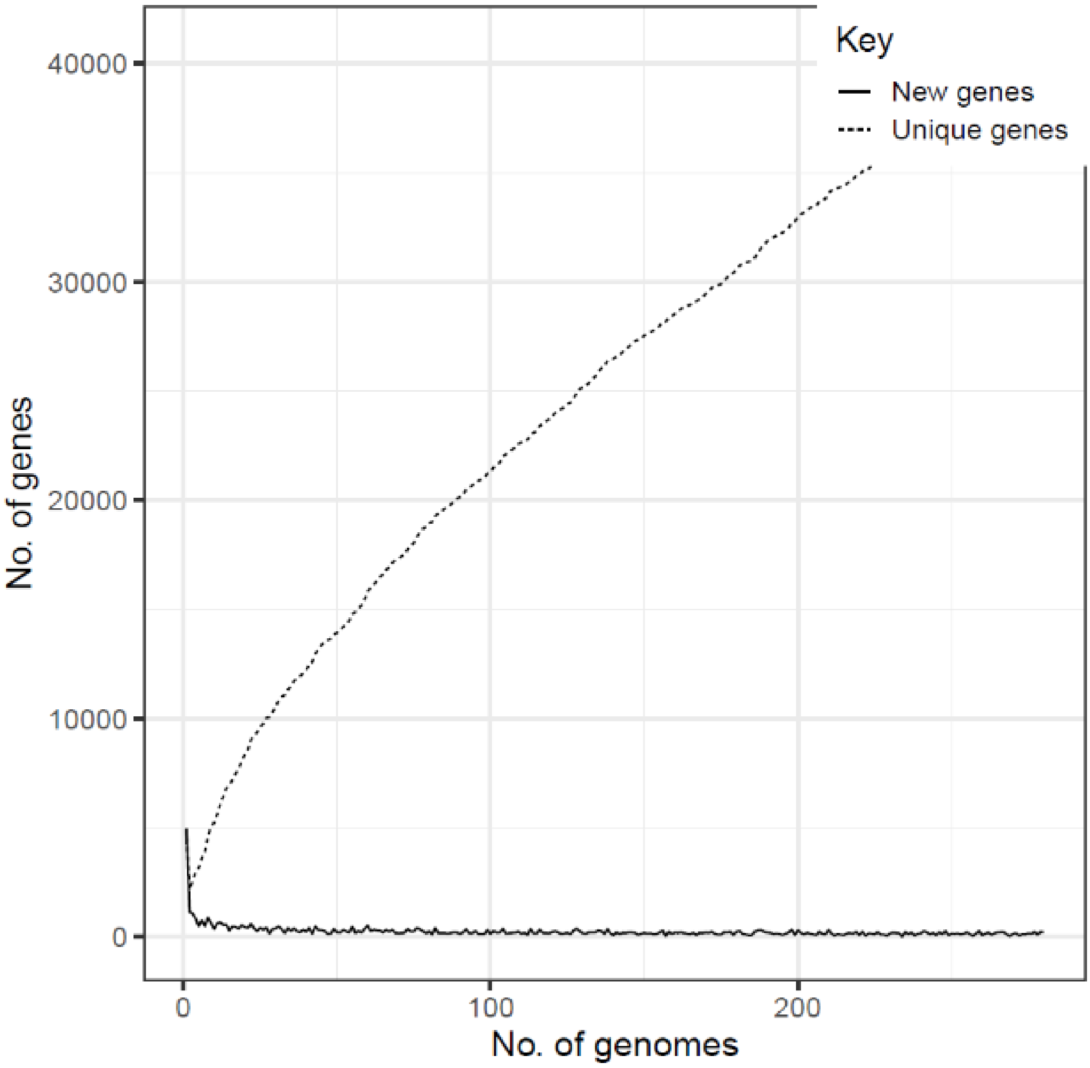

Figure S2

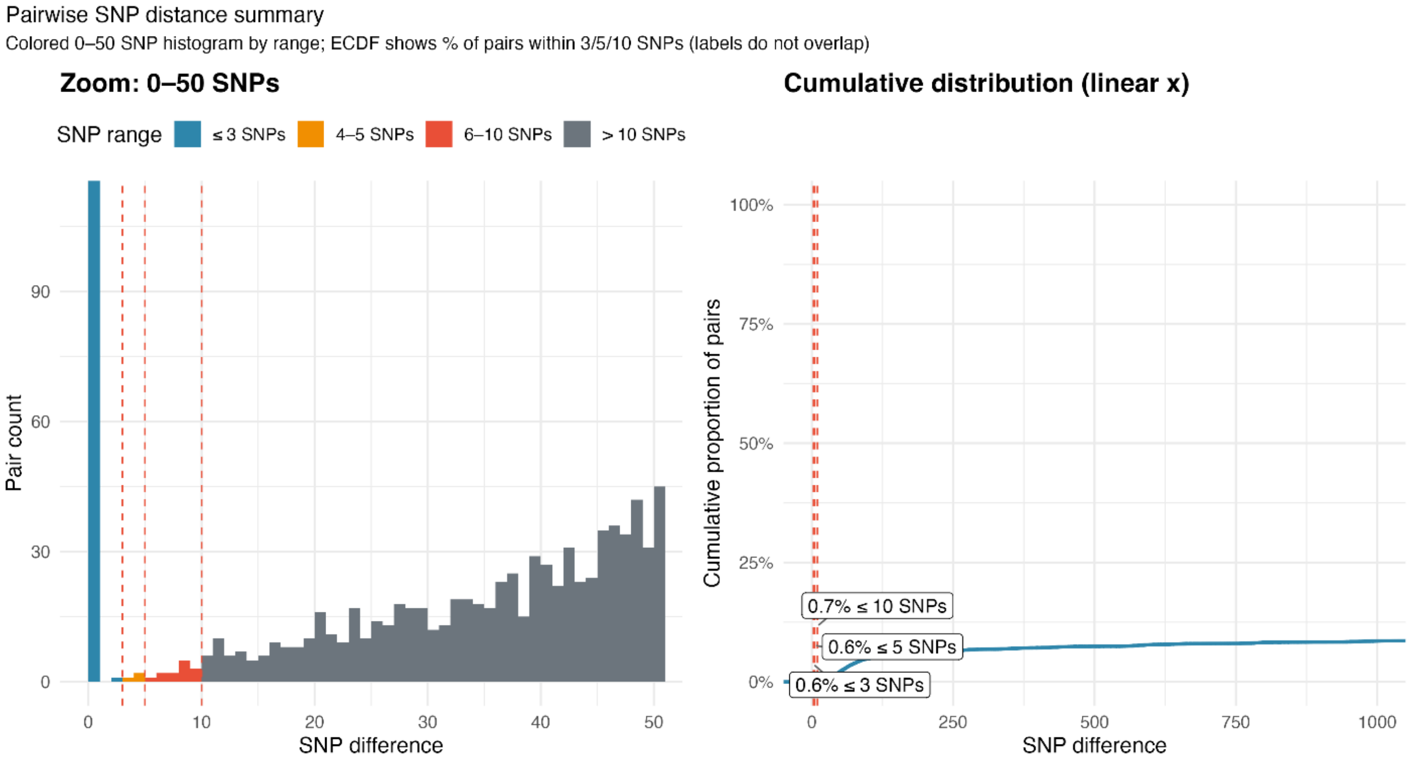
Pairwise SNP distance summary
Colored 0–50 SNP histogram by range; ECDF shows % of pairs within 3/5/10 SNPs (labels do not overlap)
Zoom: 0–50 SNPs
SNP range
≤ 3 SNPs
4–5 SNPs
6–10 SNPs
> 10 SNPs
Pair count
0
30
60
90
0
10
20
30
40
50
SNP difference
Cumulative distribution (linear x)
Cumulative proportion of pairs
0%
25%
50%
75%
100%
0.7% ≤ 10 SNPs
0.6% ≤ 5 SNPs
0.6% ≤ 3 SNPs
0
250
500
750
1000
SNP difference

Figure S3

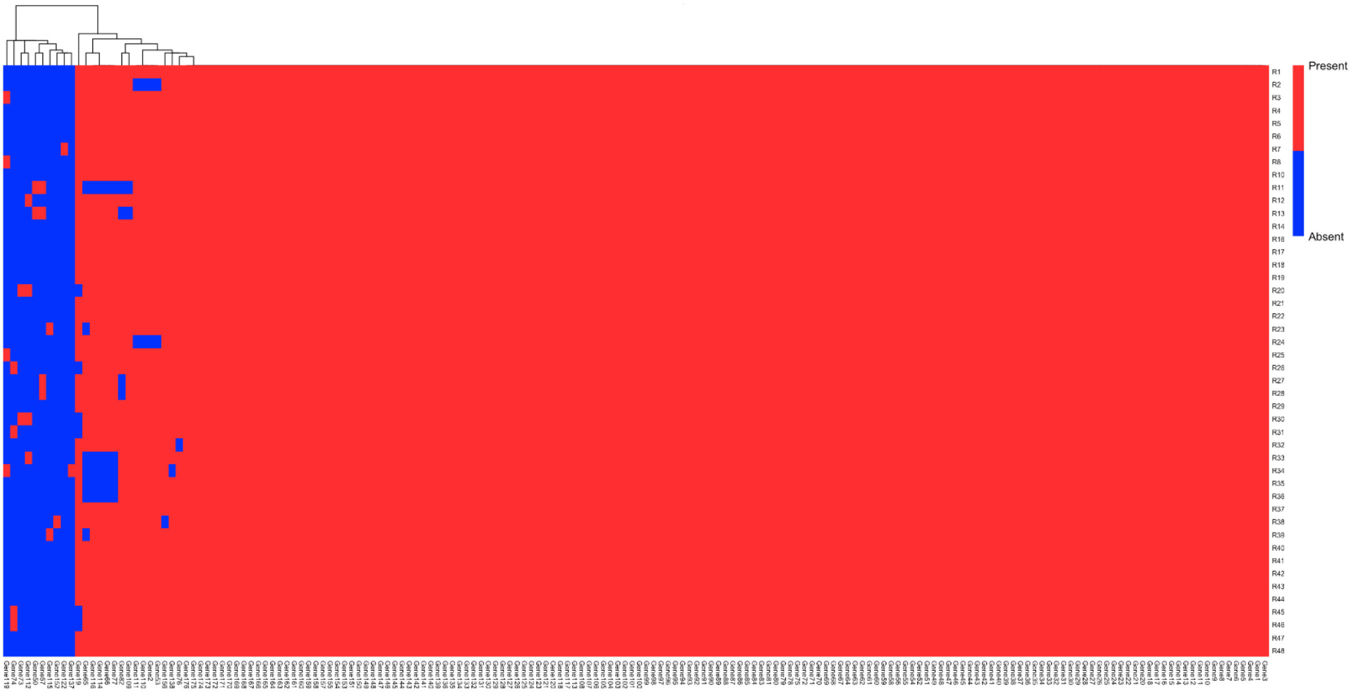

Figure S4

a

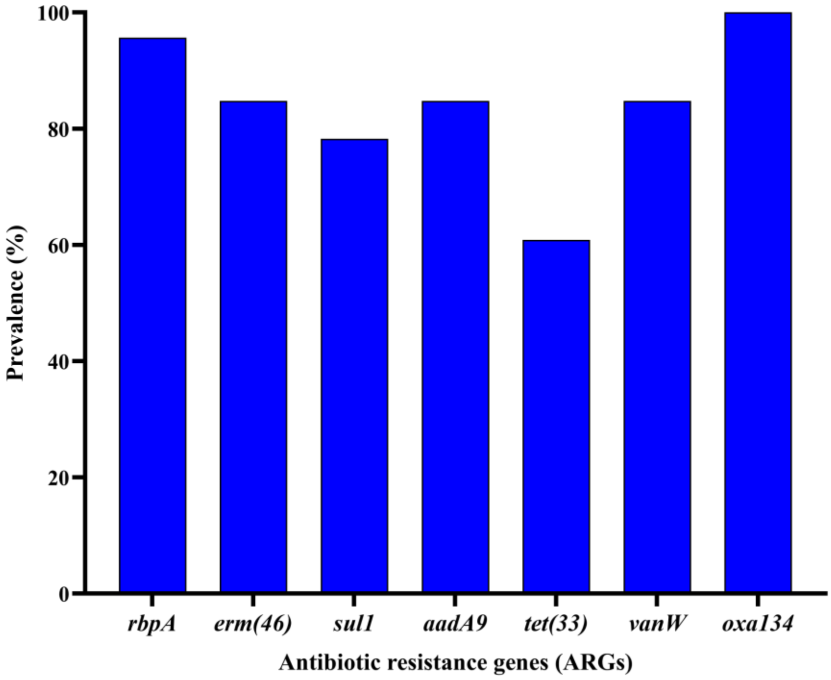

100
80
60
40
20
0
Prevalence (%)
rbpA
erm(46)
sul1
aadA9
tet(33)
vanW
oxa134
Antibiotic resistance genes (ARGs)

b

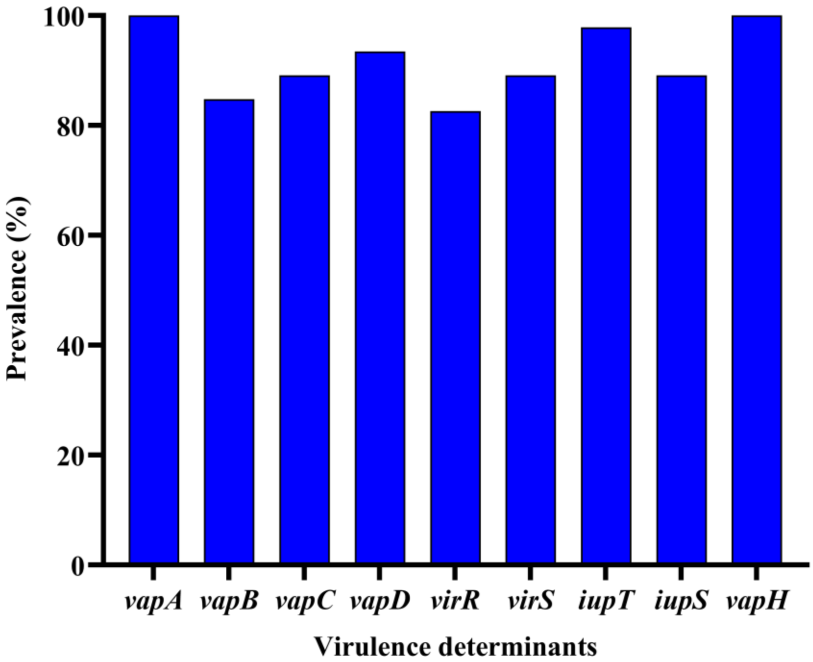

100
80
60
40
20
0
Prevalence (%)
vapA
vapB
vapC
vapD
virR
virS
iupT
iupS
vapH
Virulence determinants
