# Supplementary material for: Genomic diversity of multidrug-resistant Rhodococcus equi: novel sequence types, pangenome architecture, and phylogenomic evolution
Source: Appl Environ Microbiol. 2026 Jun 4;92(7):e02486-25. doi: 10.1128/aem.02486-25 (PMC13390474; doi:10.1128/aem.02486-25)
Supplement: Supplemental legends — Descriptive legends for supplemental material. [file aem.02486-25-s0004.docx]

**Figure S1. (a)** The pie-chart represents the overview of the numbers and types of genes identified using pan-genome analysis. The X-axis on the graphs represents the number of genomes used to perform pan-genome analysis of 46 *R. equi* isolates obtained from horses and the Y-axis represents the number of genes detected; **(b)** Effect on the number of genes on increasing the number of genomes analyzed **(c)** Effect on number of conserved/core genes on increasing the number of genomes for analysis; and **(d)** Effect in the frequency of new and unique genes on increasing the number of genomes for pan-genome analysis.

**Figure S2.** Pairwise single nucleotide polymorphism (SNP) distances across all *R. equi* isolate pairs. **Left panel:** Histogram of SNP distances (zoomed to 0–50 SNPs) with color-coded ranges highlighting genomic thresholds. Bars representing ≤3 SNPs (blue), 4–5 SNPs (yellow), and 6–10 SNPs (orange) indicate closely related isolate pairs, while grey bars (>10 SNPs) represent unrelated background diversity. Vertical dashed lines mark the 3-, 5-, and 10-SNP cutoffs. Only a small number of isolate pairs fell within these low-SNP ranges. **Right panel:** Empirical cumulative distribution function showing cumulative proportion of isolate pairs across the full SNP distance range. Annotations indicate the proportion of isolate pairs within ≤3, ≤5, and ≤10 SNPs (0.6%, 0.6%, and 0.7%, respectively).

**Figure S3.** Heatmap showing the distribution of virulence-associated genes across the 46 R. equi isolates. Each column represents an annotated virulence gene, and each row corresponds to an isolate. Red indicates gene presence, while blue indicates gene absence. Hierarchical clustering (top dendrogram) groups isolate based on similarity in their virulence gene profiles.

**Figure S4.** Bar graphs represent the prevalence of; **(a)** Antimicrobial resistance genes (ARGs), and **(b)** Virulence determinants. Both ARGs and virulence determinants were detected using PCR.

**Supplementary File 1**. Metadata and genomic information for *R. equi* genomes included in this study. This Excel file contains multiple sheets summarizing the metadata associated with the analyzed genome assemblies, including isolate identifiers, NCBI assembly accession numbers, year of isolation, source, and other relevant genomic information.

**Supplementary File 2**. Whole genome analysis of virulence factor genes. The excel file contains multiple sheets containing information about the chromosomal virulence factor genes (VRGs) as annotated in by the virulence factor database.
