# Supplementary material for: Genomic diversity of multidrug-resistant Rhodococcus equi: novel sequence types, pangenome architecture, and phylogenomic evolution
Source: Appl Environ Microbiol. 2026 Jun 4;92(7):e02486-25. doi: 10.1128/aem.02486-25 (PMC13390474; doi:10.1128/aem.02486-25)
Supplement: Supplemental tables — Tables S1 to S8. [file aem.02486-25-s0005.docx]

**Table S1.** MIC breakpoints for human associated Staphylococcus aureus provided by CLSI.

| **Antibiotics** | **Abbreviation** | **MIC breakpoints** | | |
| --- | --- | --- | --- | --- |
|  |  | **Sensitive** | **Intermediate** | **Resistant** |
| Amikacin | AMI | ≤16 |  | ≥16 |
| Ampicillin | AMP | ≤2 |  | ≥4 |
| Cefazolin | FAZ | ≤2 |  | ≥4 |
| Chloramphenicol | CHL | ≤8 | 16 | ≥32 |
| Clarithromycin | CLA | ≤2 | 4 | ≥8 |
| Doxycycline | DOX | ≤4 | 8 | ≥16 |
| Erythromycin | ERY | ≤2 | 1 to 4 | ≥8 |
| Gentamicin | GEN | ≤4 | 8 | ≥16 |
| Imipenem | IMI | ≤2 |  | ≥4 |
| Minocycline | MIN | ≤4 | 8 | ≥16 |
| Oxacillin + 2% NaCl | OXA+ | ≤2 |  | ≥4 |
| Penicillin | PEN | ≤0.12 |  | ≥0.25 |
| Rifampin | RIF | ≤1 | 2 | ≥4 |
| Tetracycline | TET | ≤4 | 8 | ≥16 |
| Trimethoprim/Sulfamethoxazole | SXT | ≤2/38 | 9 | ≥4/76 |

**Table S2:** List of virulence-associated gene primers used in this study.

| **Genes** | **Sequence 5’-3’** | **Amplicon size** | **References** |
| --- | --- | --- | --- |
| Virulence associated protein A (*vapA*) | F-GACTCTTCACAAGACGGT | 563 | (Javed et al., 2017) |
|  | R-TAGGCGTTGTGCCAGCTA |  |  |
| Virulence associated protein B (*vapB*) | F- CTTCTTAAGGATGGGGCAGG | 485 | (Bryan et al., 2018) |
|  | R- GGCTACCTTCAGCCTGCTAT |  |  |
| Virulence associated protein C (*vapC*) | F- CCGTTGTGGCGAGTGTCCTA | 441 | (Byrne et al., 2001) |
|  | R- AAGAATGCGACCGTAACTGT |  |  |
| Virulence associated protein D (*vapD*) | F- GGCGTTTATTCACTTTCTTG | 402 | (Byrne et al., 2001) |
|  | R- AGTCGTTCGCCCTTGCTGTC |  |  |
| Virulence associated protein H (*vapH*) | F-AATTCCTATCAAGGACAGC | 500 | (Monego et al., 2009) |
|  | R-ATACCGATTACGGAGCTCAC |  |  |
| Iron acquisition (*iupS*) | F- GCTGGACAGCTACACCTTCC | 234 | (Miranda-Casoluengo et al., 2012) |
|  | R- GTTGTTGACGATGCCGATGT |  |  |
| Iron acquisition (*iupT*) | F- GGGGCTGCACTACCTCAGTA | 193 | (Miranda-Casoluengo et al., 2012) |
|  | R- CTTACGCGGACGGAAACAC |  |  |
| Transcriptional regulator (*virR*) | F- CGGACGAGTTCGACTGGTAT | 450 | (Russell et al., 2004) |
|  | R- CAAAGACGATTTGGGGTACG |  |  |
| Transcriptional regulator (*virS*) | F- GAACAACTGGGAATGGTGGT | 147 | (Wang et al., 2014) |
|  | R- GTTCGCCGTTTCTAGACGAA |  |  |

**Table S3:** List of antimicrobial resistance gene primers used in this study.

| **Genes** | **Sequence 5’-3’** | **Target antibiotics** | **Amplicon size** | **References** |
| --- | --- | --- | --- | --- |
| RNA polymerase-binding protein (*RbpA*) | F-GACTCTTCACAAGACGGT | Rifampin | 211 | This study |
|  | R-TAGGCGTTGTGCCAGCTA |  |  |  |
| Erythromycin resistance methylase (*erm46)* | F-ACCTCGTTTCTCGAACAGAC | Macrolides, lincomycin, streptogramin | 363 | This study |
|  | R-ACCATTGCGCAGTCATCATC |  |  |  |
| Sulfonamide resistance gene-1 (*Sul1*) | F-TTCAATCGACAGCTTCCAAC | Sulfonamide | 439 | This study |
|  | R-ATCCTTTACAGGAAGGCCAA |  |  |  |
| Aminoglycoside adenyltransferase (*aadA9*) | F-ACGCTGAAAGCCATACACTT | Aminoglycosides | 350 | This study |
|  | R-ATGCTGTGTTGCCTAGCTTT |  |  |  |
| Efflux pumps proteins (*tet(33)*) | F-GTTCAATTCATTGGGCAGGC | Tetracyclines | 437 | This study |
|  | R-TCGGCGTTGATGTAAGTGAG |  |  |  |
| Glycopeptide resistance gene cluster (*vanW*) | F-GACTCGGGCATCATCAACAA | Glycopeptide/vancomycin | 281 | This study |
|  | R-GTCTTGGTACCCCAGATGC |  |  |  |
| Oxabeta-lactamase (*Oxa134*) | F-CGATCACGTTCACCAACAAG | Carbapenem | 288 | This study |
|  | R-TCAGCTCGTTCTTGAGGTTC |  |  |  |

**Table S4.** The descriptive analysis of prevalence of R. equi in relation to age and sex of the horses, and the tissue sites from which samples were isolated.

| **Variables** | | **Number of *R. equi* positive isolates (n=46)** | **Prevalence (%)** | **Lower CI (%)** | **Upper CI (%)** | **p-value** |
| --- | --- | --- | --- | --- | --- | --- |
| **Age** | 0-6 months | 37 | 80.43 | 90.71 | 68.67 | <0.0001* |
|  | Yearling (1-2 years) | 3 | 2.17 | 11.57 | 0.11 |  |
|  | Juvenile (2-3 years) | 5 | 10.87 | 23.50 | 4.84 |  |
|  | Colts (3-4 years) | 1 | 2.17 | 11.57 | 0.11 |  |
|  | Unknown | 1 | 2.17 | 11.57 | 0.11 |  |
|  | | | | | | |
| **Sex** | Male | 17 | 36.96 | 51.40 | 24.52 | <0.0001* |
|  | Female | 24 | 52.17 | 65.88 | 38.14 |  |
|  | Unknown | 5 | 10.87 | 23.04 | 4.73 |  |
|  | | | | | | |
| **Female** | 0-6 months | 21 | 61.76 | 90.44 | 52.74 | 0.5294^ns^ |
|  | Yearlings (1-2 years) | 0 | 0.00 | 26.98 | 0.30 |  |
|  | Juvenile (2-3 years) | 3 | 60.00 | 34.34 | 2.09 |  |
|  | colt (3-4 years) | 0 | 0.00 | 26.98 | 0.30 |  |
| **Male** | 0-6 months | 13 | 38.24 | 90.44 | 52.74 | 0.5294 ^ns^ |
|  | Yearlings (1-2 years) | 1 | 100.00 | 26.98 | 0.30 |  |
|  | Juvenile (2-3 years) | 2 | 66.67 | 34.34 | 2.09 |  |
|  | colt (3-4 years) | 1 | 100.00 | 26.98 | 0.30 |  |
|  | | | | | | |
| **Isolation sites** | Feces | 1 | 2.17 | 11.34 | 0.11 | <0.0001* |
|  | Lungs | 24 | 52.17 | 65.88 | 38.14 |  |
|  | Spleen and lymph nodes | 3 | 6.52 | 17.50 | 2.24 |  |
|  | Liver and intestines | 1 | 2.17 | 11.34 | 0.11 |  |
|  | Joints/vertebrae | 5 | 10.87 | 23.04 | 4.73 |  |
|  | Abscess | 12 | 26.09 | 40.26 | 15.60 |  |

* Statistically significant (*p* < 0.05)

^ns^ Statistically not significant (*p* > 0.05)

**Table S5.** Optical density/ absorbance (OD_600_) values for biofilms of 46 *R. equi* demonstrated and their respective categories. SBP: Strong biofilm producers, MBP: Moderate biofilm producers, WBP: Weak biofilm producers, and NBP: No biofilm producers.

| **Sample ID** | **Absorbance (OD_550_)** | **SD** | **Category** |
| --- | --- | --- | --- |
| R1 | 0.484 | 0.135765 | SBP |
| R2 | 0.242 | 0.009899 | MBP |
| R3 | 0.252 | 0.002828 | MBP |
| R4 | 1.7255 | 0.119501 | SBP |
| R5 | 1.311 | 0.154149 | SBP |
| R6 | 0.144 | 0.024042 | WBP |
| R7 | 0.237 | 0.100409 | MBP |
| R8 | 0.31 | 0.156978 | MBP |
| R10 | 0.1805 | 0.006364 | WBP |
| R11 | 0.1935 | 0.006364 | WBP |
| R12 | 0.218 | 0.009899 | MBP |
| R13 | 0.1665 | 0.013435 | WBP |
| R14 | 0.086 | 0.001414 | NBP |
| R16 | 0.195 | 0.032527 | WBP |
| R17 | 0.773 | 0.173948 | SBP |
| R18 | 0.1385 | 0.007778 | WBP |
| R19 | 0.1555 | 0.03182 | WBP |
| R20 | 0.2385 | 0.108187 | MBP |
| R21 | 0.1165 | 0.007778 | WBP |
| R22 | 0.146 | 0.002828 | WBP |
| R23 | 0.771 | 0.543058 | SBP |
| R24 | 0.4465 | 0.136472 | SBP |
| R25 | 2.0475 | 0.024749 | SBP |
| R26 | 0.3295 | 0.04879 | MBP |
| R27 | 0.1915 | 0.028991 | WBP |
| R28 | 0.1355 | 0.026163 | WBP |
| R29 | 0.1435 | 0.017678 | WBP |
| R30 | 0.2155 | 0.012021 | MBP |
| R31 | 0.175 | 0.012728 | WBP |
| R32 | 2.105 | 2.648822 | SBP |
| R33 | 0.4885 | 0.221324 | SBP |
| R34 | 0.126 | 0 | WBP |
| R35 | 0.131 | 0.002828 | WBP |
| R36 | 2.617 | 0.36911 | SBP |
| R37 | 0.1415 | 0.003536 | WBP |
| R38 | 0.148 | 0.008485 | WBP |
| R39 | 0.1675 | 0.013435 | WBP |
| R40 | 0.955 | 0.181019 | SBP |
| R41 | 2.084 | 0.684479 | SBP |
| R42 | 0.516 | 0.089095 | SBP |
| R43 | 0.2165 | 0.009192 | MBP |
| R44 | 0.1745 | 0.007778 | WBP |
| R45 | 0.154 | 0.004243 | WBP |
| R46 | 0.156 | 0.011314 | WBP |
| R47 | 0.1635 | 0.010607 | WBP |
| R48 | 0.155 | 0.014142 | WBP |

**Table S6.** Mean CFU/mL with standard deviation (SD) representing survival of 46 R. equi isolates from horses in murine macrophage cell lines.

| **Sample ID** | **Mean CFU/mL** | **SD** |
| --- | --- | --- |
| R1 | 1.60E+05 | 1.63E+04 |
| R2 | 3.50E+06 | 1.00E+06 |
| R3 | 8.50E+06 | 3.00E+06 |
| R4 | 1.60E+07 | 2.83E+06 |
| R5 | 7.50E+04 | 1.00E+04 |
| R6 | 5.50E+04 | 1.91E+04 |
| R7 | 4.00E+06 | 1.63E+06 |
| R8 | 3.50E+06 | 1.00E+06 |
| R10 | 9.00E+04 | 3.46E+04 |
| R11 | 1.15E+05 | 2.52E+04 |
| R12 | 1.20E+05 | 2.83E+04 |
| R13 | 7.00E+04 | 2.00E+04 |
| R14 | 4.50E+04 | 1.00E+04 |
| R16 | 6.00E+04 | 1.63E+04 |
| R17 | 1.45E+07 | 3.00E+06 |
| R18 | 9.00E+04 | 1.15E+04 |
| R19 | 1.15E+05 | 1.91E+04 |
| R20 | 7.00E+04 | 2.00E+04 |
| R21 | 5.50E+06 | 1.00E+06 |
| R22 | 4.00E+06 | 1.63E+06 |
| R23 | 6.00E+04 | 1.63E+04 |
| R24 | 1.00E+05 | 2.83E+04 |
| R25 | 5.50E+04 | 1.00E+04 |
| R26 | 4.00E+06 | 1.63E+06 |
| R27 | 5.50E+06 | 3.00E+06 |
| R28 | 2.35E+05 | 5.51E+04 |
| R29 | 1.55E+05 | 1.00E+04 |
| R30 | 2.05E+05 | 4.73E+04 |
| R31 | 7.50E+06 | 4.12E+06 |
| R32 | 1.15E+05 | 1.91E+04 |
| R33 | 5.50E+04 | 1.91E+04 |
| R34 | 1.35E+05 | 3.42E+04 |
| R35 | 3.50E+06 | 1.00E+06 |
| R36 | 3.20E+07 | 2.83E+06 |
| R37 | 2.15E+05 | 3.42E+04 |
| R38 | 1.65E+05 | 3.79E+04 |
| R39 | 2.10E+05 | 2.58E+04 |
| R40 | 2.00E+05 | 2.83E+04 |
| R41 | 2.15E+05 | 3.42E+04 |
| R42 | 1.00E+05 | 3.27E+04 |
| R43 | 2.40E+05 | 4.32E+04 |
| R44 | 1.65E+05 | 3.00E+04 |
| R45 | 2.45E+05 | 4.43E+04 |
| R46 | 2.25E+05 | 5.00E+04 |
| R47 | 3.50E+05 | 5.29E+04 |
| R48 | 9.50E+04 | 6.19E+04 |
| ***R equi* 103s** | 3.20E+07 | 2.83E+06 |

**Table S7**. Multi-locus Sequencing Typing (MLST) of 46 R. equi isolates collected from horses.

| Sequence types (MLST) | No. of isolates | Isolates | Type |
| --- | --- | --- | --- |
| ST-2 | 4 | R26, R31, R45, R46 | Previously Reported |
| ST-16 | 2 | R3, R25 | Previously Reported |
| ST-83 | 7 | R1, R4, R14, R17, R18, R32, R48 | Novel |
| ST-84 | 6 | R5, R10, R12, R22, R29, R47 | Novel |
| ST-85 | 2 | R11, R13 | Novel |
| ST-86 | 6 | R6, R16, R35, R36, R37, R42 | Novel |
| ST-87 | 1 | R19 | Novel |
| ST-88 | 2 | R2, R24 | Novel |
| ST-89 | 2 | R20, R30 | Novel |
| ST-90 | 2 | R21, R41 | Novel |
| ST-91 | 1 | R23 | Novel |
| ST-92 | 1 | R27 | Novel |
| ST-93 | 1 | R28 | Novel |
| ST-94 | 1 | R33 | Novel |
| ST-95 | 1 | R34 | Novel |
| ST-96 | 1 | R38 | Novel |
| ST-97 | 1 | R39 | Novel |
| ST-98 | 1 | R40 | Novel |
| ST-99 | 1 | R43 | Novel |
| ST-100 | 1 | R44 | Novel |
| ST-101 | 1 | R7 | Novel |
| ST-102 | 1 | R8 | Novel |

**Table S8.** Whole genome sequence prediction of antibiotic resistance determinants with the overall coverage percentage. N/A represents genes not identified or detected

| **Sample ID** | **Genes Identified with sequence coverage (%)** | | | | | | **No. of genes identified** |
| --- | --- | --- | --- | --- | --- | --- | --- |
|  | ***rpoB2*** | ***rbpA*** | ***aadA9*** | ***erm(46)*** | ***sul1*** | ***tet(33)*** |  |
| R1 | 98.6 | 96.52 | N/A | 100 | N/A | N/A | 3 |
| R2 | 98.6 | 96.52 | N/A | N/A | N/A | N/A | 2 |
| R3 | 98.6 | 96.52 | N/A | N/A | N/A | N/A | 2 |
| R4 | 98.6 | 96.52 | 100 | 100 | 100 | 100 | 6 |
| R5 | 98.6 | 96.52 | N/A | N/A | N/A | N/A | 2 |
| R6 | 98.6 | 96.52 | N/A | 100 | N/A | N/A | 3 |
| R7 | 98.6 | 96.52 | N/A | N/A | N/A | N/A | 2 |
| R8 | 98.6 | 96.52 | N/A | N/A | N/A | N/A | 2 |
| R10 | 98.6 | 96.52 | N/A | N/A | N/A | N/A | 2 |
| R11 | 98.6 | 96.52 | N/A | N/A | N/A | N/A | 2 |
| R12 | 98.6 | 96.52 | N/A | N/A | N/A | N/A | 2 |
| R13 | 98.6 | 96.52 | N/A | N/A | N/A | N/A | 2 |
| R14 | 98.6 | 96.52 | 100 | 100 | 99.76 | 100 | 6 |
| R16 | 98.6 | 96.52 | N/A | N/A | N/A | N/A | 2 |
| R17 | 98.6 | 96.52 | N/A | N/A | N/A | N/A | 2 |
| R18 | 98.6 | 96.52 | 100 | 100 | 100 | 100 | 6 |
| R19 | 98.6 | 96.52 | N/A | N/A | N/A | N/A | 2 |
| R20 | 98.6 | 96.52 | N/A | N/A | N/A | N/A | 2 |
| R21 | 98.6 | 96.52 | N/A | N/A | N/A | N/A | 2 |
| R22 | 98.6 | 96.52 | N/A | N/A | N/A | N/A | 2 |
| R23 | 98.6 | 96.52 | N/A | N/A | N/A | N/A | 2 |
| R24 | 98.6 | 96.52 | N/A | N/A | N/A | N/A | 2 |
| R25 | 98.6 | 96.52 | N/A | N/A | N/A | N/A | 2 |
| R26 | 98.6 | 96.52 | N/A | N/A | N/A | N/A | 2 |
| R27 | 98.6 | 96.52 | N/A | N/A | N/A | N/A | 2 |
| R28 | 98.6 | 96.52 | N/A | N/A | N/A | N/A | 2 |
| R29 | 98.6 | 96.52 | N/A | N/A | N/A | N/A | 2 |
| R30 | 98.6 | 96.52 | N/A | N/A | N/A | N/A | 2 |
| R31 | 98.6 | 96.52 | N/A | N/A | N/A | N/A | 2 |
| R32 | 98.6 | 96.52 | N/A | 98.52 | N/A | N/A | 3 |
| R33 | 98.6 | 96.52 | N/A | N/A | N/A | N/A | 2 |
| R34 | 98.6 | 96.52 | N/A | N/A | N/A | N/A | 2 |
| R35 | 98.6 | 96.52 | N/A | 100 | N/A | N/A | 3 |
| R36 | 98.6 | 96.52 | N/A | 100 | N/A | N/A | 3 |
| R37 | 98.6 | 96.52 | 100 | 100 | 100 | 100 | 6 |
| R38 | 98.6 | 96.52 | N/A | N/A | N/A | N/A | 2 |
| R39 | 98.6 | 96.52 | N/A | N/A | N/A | N/A | 2 |
| R40 | 98.6 | 96.52 | N/A | N/A | N/A | N/A | 2 |
| R41 | 98.6 | 96.52 | N/A | N/A | N/A | N/A | 2 |
| R42 | 98.6 | 96.52 | N/A | N/A | N/A | N/A | 2 |
| R43 | 98.6 | 96.52 | N/A | N/A | N/A | N/A | 2 |
| R44 | 98.6 | 96.52 | N/A | N/A | N/A | N/A | 2 |
| R45 | 98.6 | 96.52 | N/A | N/A | N/A | N/A | 2 |
| R46 | 98.6 | 96.52 | N/A | N/A | N/A | N/A | 2 |
| R47 | 98.6 | 96.52 | N/A | N/A | N/A | N/A | 2 |
| R48 | 98.6 | 96.52 | N/A | 100 | N/A | N/A | 3 |

**References:**

Bryan, L. K., Alexander, E. R., Lawhon, S. D., & Cohen, N. D. (2018). Detection of vapN in Rhodococcus equi isolates cultured from humans. *PLOS ONE*, *13*(1), e0190829. <https://doi.org/10.1371/journal.pone.0190829>

Byrne, B. A., Prescott, J. F., Palmer, G. H., Takai, S., Nicholson, V. M., Alperin, D. C., & Hines, S. A. (2001). Virulence plasmid of Rhodococcus equi contains inducible gene family encoding secreted proteins. *Infect Immun*, *69*(2), 650-656. <https://doi.org/10.1128/iai.69.2.650-656.2001>

Javed, R., Taku, A. K., Sharma, R. K., & Badroo, G. A. (2017). Molecular characterization of Rhodococcus equi isolates in equines. *Vet World*, *10*(1), 6-10. <https://doi.org/10.14202/vetworld.2017.6-10>

Miranda-Casoluengo, R., Coulson, G. B., Miranda-Casoluengo, A., Vázquez-Boland, J. A., Hondalus, M. K., & Meijer, W. G. (2012). The hydroxamate siderophore rhequichelin is required for virulence of the pathogenic actinomycete Rhodococcus equi. *Infect Immun*, *80*(12), 4106-4114. <https://doi.org/10.1128/iai.00678-12>

Monego, F., Maboni, F., Krewer, C., Vargas, A., Costa, M., & Loreto, E. (2009). Molecular Characterization of Rhodococcus equi from Horse-Breeding Farms by Means of Multiplex PCR for the vap Gene Family. *Current Microbiology*, *58*(4), 399-403. <https://doi.org/10.1007/s00284-009-9370-6>

Russell, D. A., Byrne, G. A., O'Connell, E. P., Boland, C. A., & Meijer, W. G. (2004). The LysR-type transcriptional regulator VirR is required for expression of the virulence gene vapA of Rhodococcus equi ATCC 33701. *J Bacteriol*, *186*(17), 5576-5584. <https://doi.org/10.1128/jb.186.17.5576-5584.2004>

Wang, X., Coulson, G. B., Miranda-Casoluengo, A. A., Miranda-Casoluengo, R., Hondalus, M. K., & Meijer, W. G. (2014). IcgA is a virulence factor of Rhodococcus equi that modulates intracellular growth. *Infect Immun*, *82*(5), 1793-1800. <https://doi.org/10.1128/iai.01670-13>
